# Parenting behaviors over time and their effects on cortical and limbic brain structure in children and young adults

**DOI:** 10.1016/j.dcn.2026.101728

**Published:** 2026-04-19

**Authors:** Mirjam Habegger, Plamina Dimanova, Réka Borbás, Elena Federici, Denis Ribeaud, Manuel P. Eisner, Todd A. Hare, Nora Maria Raschle

**Affiliations:** a Jacobs Center for Productive Youth Development at the University of Zurich, Switzerland; b Zurich Center for Neuroeconomics, Department of Economics, University of Zurich, Switzerland; c Neuroscience Center Zurich, University of Zurich and ETH Zurich, Switzerland; d Violence Research Center, Institute of Criminology, University of Cambridge, UK

**Keywords:** Parenting behaviors, Brain, Development, Corticolimbic brain structure, Emotion processing, Emotion regulation

## Abstract

Parenting behaviors significantly impact children's development, including maturation of emotion regulation skills and associated brain regions (e.g., amygdala, hippocampus, prefrontal cortex). Adaptive and maladaptive parenting behaviors have been associated with brain structure of children/adolescents; however, longitudinal studies of parenting behaviors and their links to neural correlates remain scarce. Two community cohorts were analyzed: cohort 1 (N = 40) with childhood parenting measures and neuroimaging, cohort 2 with repeated-measures parenting reports from childhood to adolescence (ages 7–17; N = 1482) and neuroimaging at age 22 (n = 134). Changes in parenting behaviors, parent-child alignment at age 11, and associations between early parenting behaviors and brain structure (volume/cortical thickness) in childhood (cohort 1) and late adolescence/young adulthood (cohort 2) were assessed using linear mixed models, regressions and correlations. Results show that adaptive parenting (involvement, positive parenting) and most maladaptive behaviors (inconsistent discipline, corporal punishment) decreased with children’s age, while poor monitoring increased. Parent-child reports at age 11 were positively correlated. Positive parenting behaviors in childhood were associated with larger amygdala volume in children but smaller amygdala volume in a matched subgroup of late adolescents/young adults. Corporal punishment was associated with reduced left dorsolateral prefrontal thickness in children. These associations were robust to adjustment for multiple potential confounders, including parental and child health. In conclusion, consistent with past evidence, adaptive parenting behaviors showed developmentally specific associations with limbic brain structures; maladaptive parenting behaviors were associated with alterations in prefrontal brain structure during childhood. Our findings indicate that variations in positive parenting are associated with neurodevelopment in an age-dependent manner.

## 1 Introduction

Parental behaviors play a crucial role in children’s psychosocial and biological development. Parenting behaviors can, for example, influence the development of children’s emotion processing and regulation skills (Zimmer-Gembeck et al., 2022) by means of emotion socialization (Eisenberg et al., 1998), thus allowing appropriate management of emotional responses (Gross, 2002). Parenting behaviors can be categorized into adaptive or maladaptive practices based on the expected direction of influence of these behaviors on a child’s development (Eisenberg et al., 1998, Tan et al., 2020). Adaptive parenting behaviors, such as the display of high sensitivity or warmth, positive reinforcement (e.g., praising), consistent discipline, and parental involvement, are linked to healthy child development, including greater well-being and a reduced risk for mental health problems, such as depression (Piko and Balázs, 2012), anxiety (Butterfield et al., 2021), aggression (Kawabata et al., 2011) and disruptive behaviors (Lansford et al., 2024). In contrast, maladaptive parenting behaviors, such as maltreatment, excessive control, harshness, inconsistent discipline, poor supervision, and low warmth and sensitivity, impair children’s behavioral and psychological development and increase the risk for psychopathology (Lansford et al., 2024, Smokowski et al., 2015, Auerbach et al., 2011, MacKenzie et al., 2013).

While parents remain key players in a child’s life, their influence on children’s behavior typically decreases with age, paralleling children’s increasing autonomy and socioemotional skills (Gee, 2020, Smetana, 2011). Cross-sectional studies suggest that parents display both fewer adaptive and maladaptive parenting behaviors across childhood and adolescence (Frick et al., 1999, Shelton et al., 1996), but such designs limit inferences about true developmental change (Menard, 2002). Longitudinal studies report short-term stability of most parenting behaviors (Frick et al., 1999, Essau et al., 2006, Gross et al., 2017), while emerging evidence from longer follow-ups indicate more heterogenous trajectories, including linear declines and (inverted) U-shaped patterns across development (Lansford et al., 2021, Keijsers et al., 2009). Importantly, reported parenting behaviors vary by informant, with greater alignment for some behaviors but larger discrepancies for others, such as poor monitoring (Shelton et al., 1996, Florean et al., 2022, Russell et al., 2016).

Neurally, socioemotional skills such as emotion processing and regulation are supported by the maturation of corticolimbic brain regions (Ochsner et al., 2012, Zilverstand et al., 2017). Corticolimbic regions include subcortical areas of the limbic system, commonly linked to emotional reactivity (e.g., amygdala and hippocampus) as well as neocortical regions, such as the prefrontal cortex (PFC), which are implicated in higher-order regulatory functions (Ochsner et al., 2012, Zilverstand et al., 2017). While subcortical limbic regions typically reach maturational peaks relatively early in development (Uematsu et al., 2012), neocortical regions, including the PFC, undergo prolonged structural maturation across adolescence and into young adulthood, reflected in age-related reductions in grey matter volume (GMV) and cortical thickness (CT) (Mills et al., 2016, Tamnes et al., 2017). This extended developmental window suggests heightened sensitivity of corticolimbic circuits to environmental influences, including parenting behaviors, during childhood and adolescence.

Parenting behaviors have been shown to influence the maturation of corticolimbic brain structure (for reviews, see Tan et al., 2020; Belsky and de Haan, 2011). While adaptive parenting behaviors generally support healthy corticolimbic development, maladaptive practices have been linked to altered development of cortical (e.g., PFC) and subcortical (i.e., amygdala, hippocampus) regions (Buss et al., 2007, Edmiston et al., 2011, Hanson et al., 2015, Kok et al., 2015, Lee et al., 2019, Little et al., 2015, Luby et al., 2012, Luby et al., 2016, Lyons-Ruth et al., 2023, Schneider et al., 2012, Stepanous et al., 2023, Whittle et al., 2014, Hanson et al., 2010). However, findings regarding the direction of these associations remain inconsistent (Edmiston et al., 2011, Hanson et al., 2015, Bernier et al., 2019, Narita et al., 2010, Whittle et al., 2009, Kim et al., 2010). Such discrepancies may stem from differences in study design, developmental timing, and the use of prospective versus retrospective assessments of parenting behaviors (Tan et al., 2020, Belsky and de Haan, 2011, Whittle et al., 2016). Research has predominantly focused on extreme forms of maladaptive parenting, such as maltreatment (Delaney et al., 2023), which have been linked to altered neurodevelopmental trajectories, including delayed or accelerated maturation as proposed by the stress acceleration hypothesis (Callaghan and Tottenham, 2016, Tottenham, 2020). More recent work, however, highlights the importance of investigating variability in normative-range parenting behaviors and their association with neurodevelopment (Tan et al., 2020, Belsky and de Haan, 2011, Farber et al., 2022).

**Fig. 1:**
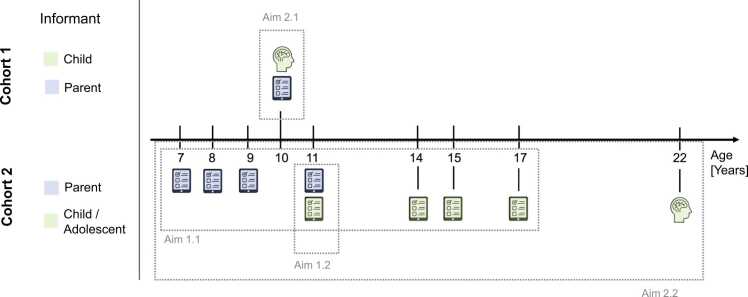
Study cohorts, design and aims. Cohort 1 includes cross-sectional behavioral and structural MRI data of 40 children and their parents, when children were aged 7–14 years (average age: 10 years). Cohort 2 consists of prospective longitudinal data, with behavioral parenting reports available at 7 time points from childhood to adolescence (N = 1482; behavioral reports from children/adolescents 7–17 years of age). Additionally, neuroimaging at 22 years of age was conducted in a subgroup of these participants (n = 134). With these two cohorts we wanted to examine characteristics of parenting behaviors and associations between parenting behaviors and corticolimbic brain structures. Specifically, variability of parenting behaviors over time (aim 1.1) and alignment between parental and adolescents’ reports (aim 1.2) was tested and the association between early parenting behaviors (received at age 7–14 years) and brain structures during childhood (aim 2.1) and late adolescence/young adulthood (2.2).

Overall, prospective longitudinal studies in community samples assessing changes in parenting behaviors and neural correlates of parenting behaviors remain scarce, particularly those including parental and child reports alongside neuroimaging data. The present study leverages data from two complementary community cohorts: one including cross-sectional behavioral and neuroimaging data collected during childhood (cohort 1) and another comprising longitudinal parenting assessments spanning 15 years with neuroimaging acquired in late adolescence/young adulthood (cohort 2; Fig. 1).

**Fig. 2:**
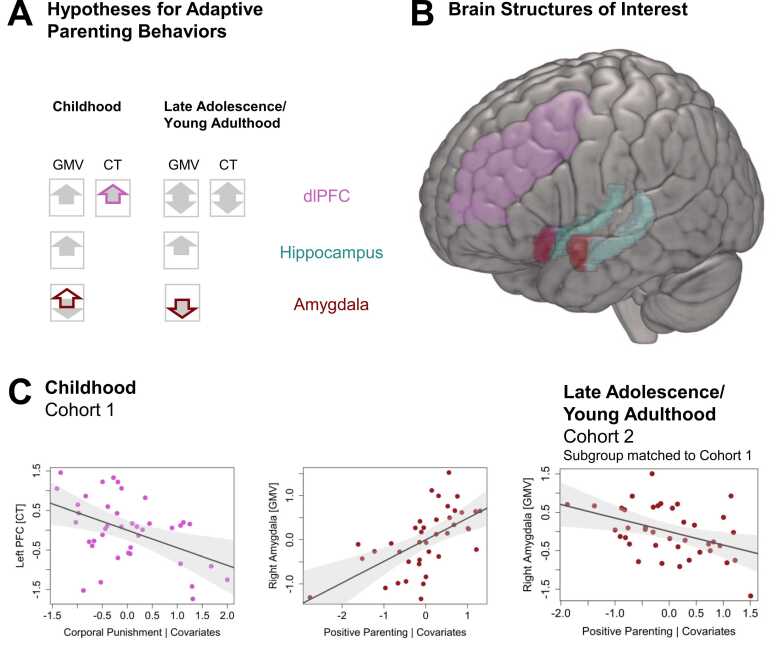
Expected and observed associations between APQ parenting behaviors and brain structures of interest. **A)** Solid (grey) arrows indicate the expected effects of adaptive parenting behaviors (i.e., positive parenting and involvement) on children’s **(aim 2.1**) and young adults’ (**aim 2.2**) GMV and CT measures of the emotion regulation brain structures as based on previous literature (Buss et al., 2007, Kok et al., 2015, Lee et al., 2019, Little et al., 2015, Luby et al., 2012, Luby et al., 2016, Lyons-Ruth et al., 2023, Schneider et al., 2012, Stepanous et al., 2023, Whittle et al., 2014, Narita et al., 2010, Kim et al., 2010). Arrow directions indicate the expected directions of association: up = positive, down = negative, up and down = evidence on both directions with primary hypotheses on negative associations. Colored arrow outline indicate the effects found in the present study. **B)** Regions of interest: bilateral dlPFC (middle frontal gyri; pink), hippocampi (turquoise), and amygdalae (red). **C)** Significant findings identified by the present study for individual APQ scales visualized by partial regression plots: The respective parenting behavior is plotted against the respective brain structure given the covariates (sex, TIV [for GMV measures], age [for cohort 1 and matched cohort 2], and all other APQ parenting behaviors) with 95% confidence intervals. APQ = Alabama Parenting Questionnaire; GMV = grey matter volume; CT = cortical thickness; dlPFC = dorsolateral prefrontal cortex.

First, we aimed to characterize age-related changes in specific parenting behaviors across childhood and adolescence using repeated measures from cohort 2 (**aim 1.1**) and to examine the alignment between children’s and parents’ reports at age 11, when data from both informants were available (**aim 1.2**; cohort 2). We hypothesized that parenting behaviors would decrease with increasing child age, and that children’s and parents’ ratings would significantly align. Second, we examined concurrent (**aim 2.1**; cohort 1) and long-term (**aim 2.2**; cohort 2) associations between parenting behaviors reported during childhood and emotion regulatory brain structures assessed during childhood and late adolescence/young adulthood, focusing on amygdala and hippocampal GMV as well as dorsolateral prefrontal cortex (dlPFC) GMV and CT. Based on prior literature (Buss et al., 2007, Kok et al., 2015, Lee et al., 2019, Little et al., 2015, Luby et al., 2012, Luby et al., 2016, Lyons-Ruth et al., 2023, Schneider et al., 2012, Stepanous et al., 2023, Whittle et al., 2014), we hypothesized that adaptive parenting behaviors would be associated with larger hippocampal GMV and dlPFC GMV/CT, but smaller amygdala GMV during childhood (Fig. 2**A**). Opposite patterns were expected for maladaptive parenting behaviors. Adaptive parenting during childhood was further expected to be associated with larger hippocampal GMV and smaller amygdala GMV in late adolescence/young adulthood, whereas maladaptive behaviors were expected to show the opposite effects. While previous reports on the direction of early parenting effects on later prefrontal development are mixed (Narita et al., 2010, Kim et al., 2010, Yang et al., 2018), we based our hypotheses on studies implicating prolonged neocortical thinning occurring into young adulthood (Tamnes et al., 2017) and findings suggesting that adaptive parenting may support these maturational processes. Consequently, we hypothesized that adaptive parenting would be associated with smaller dlPFC GMV and reduced CT in late adolescence/young adulthood whereas maladaptive parenting would be associated with larger GMV and CT (Tan et al., 2020, Whittle et al., 2014). Nevertheless, we explicitly acknowledge the possibility of opposite effects, given inconsistencies in the existing literature (Narita et al., 2010, Kim et al., 2010, Yang et al., 2018).

## 2 Methods

### 2.1 Participants and group demographics

Data from two separate cohorts were analyzed because they include highly comparable parenting assessments and neuroimaging measures, enabling examination of parenting-brain associations across distinct developmental stages using existing community data. This included cross-sectional data collected during childhood from *cohort 1* (N = 40 children; mean age=10 y) (Borbás et al., 2021), allowing assessments of concurrent associations between parenting behaviors and brain structure during childhood. Additionally, longitudinal prospective data from *cohort 2,* including repeated parenting assessments from childhood to adolescence (N = 1482; ages 7–17; Fig. 1**)**, enabled examination of age-related parenting behaviors as well as associations between early parenting behavior and later brain structure, leveraging the availability of neuroimaging data in late adolescence/young adulthood (age 22 years; n = 134), and allowing evaluation of alignment between children’s and parents' ratings of parenting behaviors at age 11.

**Cohort 1 (childhood)**. All data were obtained between 2018 and 2020 (Borbás et al., 2021). According to the International Standard Classification of Education (Organisation, 1999) the families’ parental education levels (a proxy for socioeconomic status [SES]) was average or above average. Children’s cognitive abilities were assessed using a composite IQ score of the subtests Matrix Reasoning (non-verbal IQ) and Vocabulary (verbal IQ) of the fourth version of the Hamburg-Wechsler Intelligence Scale for Children (HAWIK-IV; Petermann and Petermann, 2011). All children were of average to above-average intelligence (Table 1). Parenting behaviors were reported by the mothers using the Alabama Parenting Questionnaire (APQ; Frick, 1991; Table 1). Neuroimaging data were available for 42 children recruited in Switzerland. All participants met standard MRI eligibility and safety criteria (i.e., absence of MRI contraindications). Two datasets were excluded due to a substantial developmental delay in one child and missing behavioral information in another, resulting in a final group of 40 children.

**Table 1:** Sociodemographic characteristics and descriptive information for key study variables.

	Cohort 1 (N = 40)		Cohort 2^a^ Wave 1 (N = 1247) Wave 5 (N = 1482)				Cohort 2: Neuroimaging Subgroup Full (N = 134)^b^ Matched (N = 40)				
*Variable*	M/n	SD/%	M/n	SD/%	M/n	SD/%	M/n	SD/%	W^c^	M/n	SD/%
**Sociodemographics**											
Sex (females)	17	43	600	48	715	48	51	38.1	2799	17	43
Age at BEH Assessment (y)	10	2.1	7	0.4	13.7	0.4	-	-	-	9.9	2.1
Age at MRI (y)	10	2.1	-	-	-	-	21.8	0.5	-	21.7	0.5
SES: Mean Parental Education (1−10)	8	2.9	5.6 (20 NA’s)	2.7 (20 NA’s)	-	-	6.7 (1 NA)	2.6 (1 NA)	3443*	8.1	2.5
IQ Child (total)^d^	110.6	11.1	-	-	-	-	-	-	-	-	-
**Parenting Behaviors**											
Involvement	3.9	0.3	4.2	0.4	3.1	0.6	4	0.3	1785.5*	3.9	0.4
Positive Parenting	4	0.5	4.2	0.5	3.2	0.6	4.1	0.5	2316	3.9	0.4
Poor Monitoring	1.7	0.5	1.3	0.3	1.9	0.5	1.4	0.3	3412*	1.7	0.5
Inconsistent Discipline	2.3	0.4	2.2	0.5	2.2	0.7	2.2	0.4	3008.5	2.4	0.6
Corporal Punishment	1.1	0.2	1.5	0.5	1.2	0.4	1.3	0.4	1434*	1.2	0.3
**Structural Brain Correlates**											
**CT (mm)**											
dlPFC rh	2.7	0.1	-	-	-	-	2.5	0.1		-	-
dlPFC lh	2.7	0.1	-	-	-	-	2.5	0.1		2.5	0.1
**GMV (mm**^**3**^**)**											
dlPFC rh	28’583	3’757	-	-	-	-	23’710	2860		-	-
dlPFC lh	27’872	3’260	-	-	-	-	23’293	2955		-	-
Hippocampus rh	4’463	503	-	-	-	-	4’509	395		-	-
Hippocampus lh	4’258	434	-	-	-	-	4’379	363		-	-
Amygdala rh	1’889	229	-	-	-	-	1’943	219		1’891	199
Amygdala lh	1’775	227	-	-	-	-	1’768	217		-	-
TIV	1.61 × 10^6^	153’517	-	-	-	-	1.49 × 10^6^	244’284		1’45 × 10^6^	239’352
**Ethnicity: mother (father)**^e^	**n**	**%**	**n**	**%**			**n**	**%**		**n**	**%**
African/ Black	-	-	29 (26)	2.3 (2.1)	-	-	4 (4)	3 (3)		1 (2)	2.5 (5)
East and South East Asian	-	-	59 (29)	4.7 (2.3)	-	-	7 (4)	5.2 (3)		4 (1)	10 (3)
Latin American	-	-	59 (26)	4.7 (2.1)	-	-	5 (3)	3.7 (2.2)		1 (0)	2.5 (0)
North African / Middle Eastern	-	-	32 (37)	2.6 (3)	-	-	2 (1)	1.5 (0.7)		1 (0)	2.5 (0)
Tamil	-	-	47 (49)	3.8 (3.9)	-	-	12 (12)	9 (9)		0 (0)	0 (0)
White (European / other Western countries)	-	-	993 (856)	79.6 (68.6)	-	-	103 (90)	76.9 (67.2)		32 (30)	80 (75)
Unknown	-	-	27 (223)	2.2 (17.9)	-	-	1 (20)	0.7 (14.9)		1 (7)	2.5 (18)

***Note:*** CP = Corporal Punishment; BEH = behavioral; MRI = magnetic resonance imaging; SES = socioeconomic status; IQ = intelligence quotient; CT = cortical thickness; dlPFC = dorsolateral prefrontal cortex; rh = right hemispheric; lh = left hemispheric; GMV = grey matter volume; TIV = total intracranial volume.

a Descriptives of all waves (W1-W7) can be seen in Table S1 (available online)

b Averaged parenting values across wave 1, wave 2, wave 3 and wave 4

c Wilcoxon rank-sum test statistics (W) between variables of cohort 1 and neuroimaging subgroup of cohort 2; *groups significantly differ (p < .05)

d Average of Wechsler Vocabulary and Matrices Tests.

e Information on ethnicity of cohort 2 derives from parental self-report. Ethnicity data were not collected for cohort 1.

**Cohort 2 (childhood to late adolescence/young adulthood).** Longitudinal prospective cohort data were drawn from *The Zurich Project on the Social Development from Childhood to Adulthood* (*z-proso*) (Ribeaud et al., 2022). The dataset included parent and child/adolescent-reported data which were collected between 2004 and 2018. Assessments began when children entered first grade of primary school in Zurich, Switzerland, at approximately 7 years of age. Up to 1247 parents (93.9% mothers) completed child behavior questionnaires (z-proso Project Team. z-Proso Handbook, 2025) repeatedly across four assessment waves (ages 7, 8, 9 and 11 years) via computer-assisted personal home interviews. Additionally, up to 1482 children and adolescents completed self-report questionnaires at ages 11, 14, 15 and 17 years in school settings with guidance from trained fieldworkers (Ribeaud et al., 2022, z-proso Project Team, 2024) (see Table S1 for behavioral scores and Table S2 for data availability across waves). Participants were drawn from the general community and thus represented a wide range of socioeconomic backgrounds, with parental income ranging from below 24’0000 to above 180’000 CHF (corresponding to ∼19’200–144’000 USD in 2004). The cohort was characterized by high ethnic diversity, with approximately 44% of participants having both parents born outside Switzerland, originating from over 80 countries (parental income, education level and ethnicity are reported in Table 1, Supplement Table S3). In 2018/2019, a subsample of 142 participants completed a neuroimaging session at age 22 years. This subsample was selected from the full cohort for a study on peer victimization and bullying that used a stratified random sampling procedure, in which participants were randomly sampled from strata defined by sex, victimization and related characteristics to ensure representation of variation across these experiences and characteristics (for more details, see Heumann et al., 2025). Of these, 134 participants were included in the present analyses based on availability of complete parenting and neuroimaging data. Participants with MRI contraindications or who could not complete scanning (e.g., due to claustrophobia) were excluded from neuroimaging analyses. Cohort 1 and the neuroimaging subgroup of cohort 2 differed significantly in parental education and some parenting behaviors (Table 1). Consequently, a matched subgroup approach was implemented in addition to the primary analyses to enhance comparability across cohorts.

**Ethics.** All research complied with national and international standards as approved by the ethics committees in Basel (cohort 1; Ethikkomission Nordwest- und Zentralschweiz) and Zurich (cohort 2; Ethics Committee at the Faculty of Arts and Social Sciences of the University of Zurich/Cantonal Ethics Committee of the Canton of Zurich or according to national regulations). Parents provided written informed consent for themselves and their children, while child participants gave verbal assent. In cohort 2, adolescents provided written assent (ages 14–15) and informed consent from ages 17 (see Ribeaud et al., 2022 for details).

### 2.2 Parenting behaviors

Parenting behaviors were assessed using the German version of the APQ (Essau et al., 2006, Frick, 1991). The APQ consists of 42 items for parenting behaviors rated on a 5-point Likert-type scale ranging from 1 (*never*) to 5 (*always*). 35 items were used to compute mean scores of two adaptive parenting scales (*involvement*, *positive parenting*) and three maladaptive parenting behavior scales (*poor monitoring*, *inconsistent discipline*, *corporal punishment*) (Frick and Dantagnan, 2005, Hosokawa and Katsura, 2017). Information on scale adaptations and internal consistencies is provided in the **Supplement** (Tables S4-S6). For group-matching purposes, involvement and positive parenting scores were averaged per person at each time point to build a composite adaptive parenting score, while poor monitoring, inconsistent discipline and corporal punishment were averaged for a composite maladaptive parenting score (Table S7). The parent-reported APQ primarily refers to the behaviors of the reporting parent; however, some items capture general child behaviors and may reflect household-level parenting practices. Child self-reports were only collected in later assessment waves of cohort 2 and referred to both caregivers in the household, when applicable.

### 2.3 Neuroimaging

Structural MRI data from both cohorts were preprocessed with FreeSurfer (v7.1.0 for cohort 1; v7.2.0 for cohort 2; https://surfer.nmr.mgh.harvard.edu/) following standard procedures. GMV was extracted for bilateral limbic regions (bilateral amygdala and hippocampus) and GMV/CT were extracted for the dlPFC, operationalized as the caudal and rostral middle frontal gyri according to the Desikan-Killiany atlas (Desikan et al., 2006). Bilateral middle frontal gyrus regions were selected as dlPFC regions of interest based on prior literature implicating this region in parenting-related modulation of emotion regulation brain networks (Kok et al., 2015, Narita et al., 2010). Details on MRI acquisition and preprocessing are provided in the Supplement. Regions of interest are illustrated in Fig. 2**B**.

### 2.4 Data analysis

When APQ items were missing, scale scores were computed by averaging all available items for that scale at the respective assessment wave. For longitudinal analyses, missing data were handled using multivariate imputation via the ‘mice’ package (Buuren and Groothuis-Oudshoorn, 2011) in R (m = 30) based on available repeated measures and child sex. Predictive mean matching was used for all parenting subscales. Longitudinal models (see next section) were run separately in each of the 30 imputed datasets, and estimates were subsequently pooled across datasets using the ‘pool()’ function in ‘mice’. Imputation parameters, diagnostic checks, and R code are provided in the Supplement (Figures S1-S2).

#### 2.4.1 Variations in parenting behaviors over time (aim 1.1)

Variations in parenting behaviors were analyzed separately for parent- and adolescent-reported data in cohort 2. To maintain consistency in adolescent reports, longitudinal models were constructed using data from ages 14–17 years, while excluding age 11 due to reduced item availability at that wave (Supplement).

Linear mixed-effects models were fitted for each parenting behavior using the ‘nlme’ package in R (v3.1–159) (Pinheiro and Bates, 2022). Each model included one parenting scale as the dependent variable, with time and sex as fixed effects and a random intercept for each participant to account for repeated measures within individuals. Time was modeled as a categorical factor to estimate contrasts (mean differences) between consecutive time points as well as the overall contrast between the first and last time point, while allowing for non-linear developmental patterns. Contrasts were obtained by refitting the same model while changing only the reference category of the time factor (i.e., for each parent-reported parenting behavior, models were fitted three times, once with age 7 as reference [for contrasts age 7–8 and 7–11], once with age 8 [for contrast 8–9] and finally with age 9 as reference level [for contrast 9–11]; for each adolescent-reported parenting beahavior, models were fitted twice, respectively). To account for unequal intervals between assessment waves, coefficients were time-adjusted to represent the annualized change by dividing the estimated mean difference by the number of years in the respective interval (see Supplementary R code). Skewed dependent variables were transformed to improve normality of residuals: involvement (squared) and positive parenting (cubed) were exponentiated, whereas poor monitoring and corporal punishment were log-transformed (ln). For child/adolescent-reported corporal punishment, the log-transformation did not substantially improve residual distributions; results for this subscale should therefore be interpreted with caution.

#### 2.4.2 Alignment of parental and children’s reports (aim 1.2)

Spearman’s rank correlations were calculated between parent- and child-reported parenting behaviors at age 11 years for cohort 2. Only items available for both informants were included. To ensure scale consistency, items from the parental responses were rescaled from 5- to the corresponding adolescents’ 4-point Likert scale by setting all values above 4 to 4 (see adaptation of the adolescent’s scale and internal consistencies; Supplement).

#### 2.4.3 Parenting behaviors and childhood brain structures (aim 2.1)

Multiple linear regression models were conducted to examine associations between parenting behaviors and brain structure. GMV of bilateral dlPFC, amygdala and hippocampus, and CT of bilateral dlPFC were entered separately as dependent variables (cohort 2). Parenting behaviors (involvement, positive parenting, poor monitoring, inconsistent discipline and corporal punishment) were included as predictors.

Regression outputs were Bonferroni corrected for multiple comparison by adjusting for the number of brain regions/measures tested (*p* < .006; *p* = .05/8). Models included sex and age as covariates, and total intracranial volume (TIV) was added for GMV analyses. Given prior evidence linking SES to both parenting behaviors (Callahan and Eyberg, 2010) and brain development (Qiu et al., 2025, Baysarowich et al., 2025), parental education (used as an SES proxy) was not included by default due to its close conceptual overlap with parenting. However, for models yielding significant effects, analyses were repeated including average parental education to assess robustness. Other SES indicators, such as household income, were not available on a usable and comparable scale across both cohorts (see details in Supplement).

#### 2.4.4 Parenting behaviors and late adolescents’/young adults’ brain structure (aim 2.2)

The same regression approach was used to assess the association between parenting behaviors during childhood and brain structure in late adolescence/young adulthood in cohort 2. Here, parent-reported parenting behavior were averaged across ages 7, 8, 9 and 11 to align with the childhood age range of cohort 1 (7–14 years) and to ensure consistency using a single informant. Stability of parenting measures across waves was assessed using intraclass correlation coefficients (ICCs) and Spearman correlations. Average-measure reliability (ICC3k) ranged from .80 to .88, indicating good reliability of the aggregated scores (Koo and Li, 2016) (Supplement Table S8 provides full stability metrics). Analyses included sex and TIV (for GMV outcomes) as covariates. Age was not included as participants were from the same school grade/age.

##### 2.4.4.1 Matched-subgroup approach

To facilitate comparability between cohort 1 and 2, a best-matched subgroup of cohort 2 was created and re-evaluated (n = 40). Matching was implemented to reduce differences in socioeconomic background and parenting exposure between cohorts and to allow a more direct comparison of parenting–brain associations across developmental stages. For each participant in cohort 2, parenting scores were selected from the assessment wave closest (within ±2 years) in age to the matched participant in cohort 1, thereby aligning developmental timing of parenting assessments across cohorts. Both cohorts were matched on parental education (as an SES proxy), sex, age and composite parenting behavior scores (adaptive and maladaptive) using the ‘find.matches’ function of the ‘Hmisc’ R-package (Harrell Jr. and Dupont, 2022) (Supplement). After matching, no significant differences were observed between cohort 1 and the matched cohort 2 subgroup in any parenting behavior or parental education. We then tested if the significant associations reported for cohort 1 were also present when linking comparable variations in parenting behaviors to brain structure in late adolescence/young adulthood within the matched subgroup of cohort 2. To this end, we applied the same linear regression approach as in the primary analyses, with parenting behaviors and covariates entered as independent variables and brain structure measures at adolescence/young adulthood age (22 years) entered as outcomes. Analyses were restricted to the same brain regions of interest (ROIs) that had shown significant associations with parenting behaviors in cohort 1, thereby limiting the number of tests and focusing on hypothesis-driven replication. P-values were Bonferroni-corrected for the number of models tested (p = .05/number of models).

## 3 Results

### 3.1 Variations of parenting behaviors over time (aim 1.1)

**Fig. 3:**
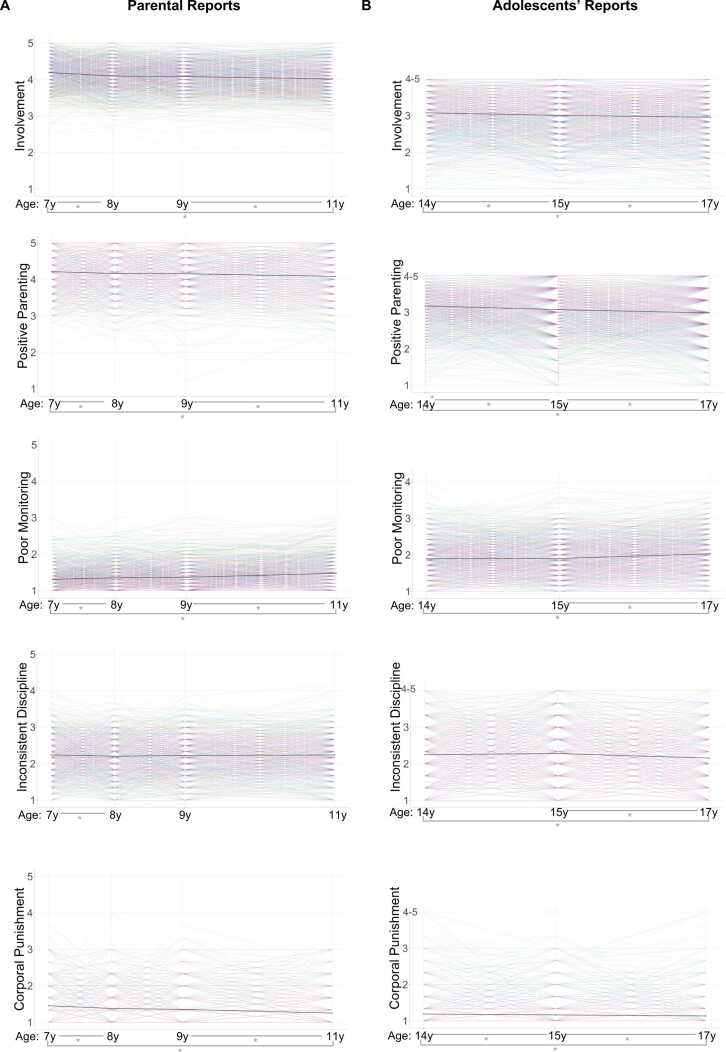
Interindividual variability and longitudinal changes of each Alabama Parenting Questionnaire parenting behavior scale for each child age reported based on **A)** parental reports and **B)** children’s/adolescents’ reports. Trajectories of each individual participant are presented in color. Group means are shown in black. Parental scores are based on items with a 5-point and children’s/adolescents’ reports on items with a 4-point Likert-type scale. Parenting behaviors show interindividual variability at each time point. *Asterisks on each panel bottom indicate significant fixed effects (decreases or increases between two assessment time points) derived from linear mixed-effect models. Significant overall decreases in parent-reported parenting behaviors were found from 7 to 11 years for involvement, positive parenting and corporal punishment, and a significant increase for poor monitoring. Significant decreases during testing from 14 to 17 years were reported for all parenting scales (i.e., involvement, positive parenting, inconsistent discipline and corporal punishment) except for poor monitoring, which showed a significant increase.

Results of the linear mixed-effect models revealed significant overall decreases in parent-reported parenting behaviors from the first to the last time point (7–11 years) in cohort 2 for involvement, positive parenting and corporal punishment, while poor monitoring significantly increased. Likewise, children/adolescents reported an overall decrease (14–17 years) in involvement, positive parenting, inconsistent discipline and corporal punishment, but an increase in poor monitoring behaviors. While overall trends were significant for most behaviors, these changes were generally not uniform across all measured intervals, suggesting developmental patterns characterized by periods of relative stability (e.g., involvement/positive parenting between ages 8 and 9) or staggered decrease/increase (inconsistent discipline and poor monitoring). An exception was corporal punishment, which showed a consistent decrease across all consecutive time points in both parent and youth reports. Table 2 and Fig. 3 depict changes between consecutive time points and individual and group average trajectories of each parenting scale.

**Table 2:** Results of linear mixed-effect models of parenting behaviors over time (aim 1.1).

	*Parental reports*					*Children’s/ Adolescents’ reports*			
	7–11 y (Overall)	7–8 y	8–9 y	9–11 y		14–17 y (Overall)	14–15 y	15–17 y	
***Parenting behaviors***	**B (*****p*****)**	**B (*****p*****)**	**B (*****p*****)**	**B (*****p*****)**	**ICC**	**B (*****p*****)**	**B (*****p*****)**	**B (*****p*****)**	**ICC**
Involvement^2^	-0.35 (<.001*)	-0.86 (<.001*)	-0.14 (.110)	-0.23 (<.001*)	0.59	-0.19 (<.001*)	-0.22 (<.001*)	-0.16 (<.001*)	0.58
Positive Parenting^3^	-1.36 (<.001*)	-2.70 (<.001*)	-0.36 (.624)	-1.26 (<.001*)	0.59	-1.49 (<.001*)	-1.63 (<.001*)	-1.36 (<.001*)	0.56
Poor Monitoring (ln)	0.03 (<.001*)	0.03 (<.001*)	0.01 (.264)	0.03 (<.001*)	0.55	0.02 (<.001*)	< 0.01 (.541)	0.03 (<.001*)	0.52
Inconsistent Discipline	0.00 (.705)	-0.04 (.014*)	0.02 (.193)	0.01 (.470)	0.52	-0.02 (<.001*)	0.02 (.111)	-0.06 (<.001*)	0.37
Corporal Punishment (ln)	-0.04 (<.001*)	-0.05 (<.001*)	-0.02 (.005*)	-0.03 (<.001*)	0.59	-0.01 (<.001*)	-0.01 (.023*)	-0.01 (.008*)	0.45

***Note:*** The first column of each informant’s reports shows overall effects from first to last time point, while the other columns show effects of consecutive time points. B shows the coefficients per year accounted for different time intervals. *P*-values of corporal punishment models need to be interpreted with caution since model assumptions (normal distribution of residuals visually inspected in qq-plots) were not met for these models. ICC = intraclass correlation coefficient. All models are corrected for sex. N = 1247 (parental reports) and N = 1482 (children’s/adolescents’ reports). * p < .05

### 3.2 Alignment of parental and children’s reports (aim 1.2)

Parental and children’s ratings at age 11 correlated positively in cohort 2 for all parenting behaviors (involvement: *r*_*s*_ = .18, *p* < .001, n = 1026; positive parenting: *r*_*s*_ = .12, *p* <.001, n = 1025; poor monitoring: *r*_*s*_ = .27, *p* < .001, n = 1025; inconsistent discipline: *r*_*s*_ = .08, *p* = .012, n = 1021; corporal punishment: *r*_*s*_ = .26, *p* < .001, n = 1025).

### 3.3 Parenting behaviors and childhood brain structures (aim 2.1)

Results of the multiple linear regression models demonstrated that positive parenting during childhood is associated with significantly larger right amygdala GMV (cohort 1). Corporal punishment is associated with significantly smaller left dlPFC CT (Table 3**,** Fig. 2**C**). These effects survived correction for multiple comparison. When adding parental education to the models, results remained similar (Table S9). See Table S10 and Figure S4 for variance inflation factors (all < 2) and correlation coefficients of all regressors.

**Table 3:** Regression results of parenting behaviors on corticolimbic brain structure.

*Variables*	β	(95% CI)	t	*p*	R^*2*^_adj_	β	(95% CI)	t	*p*	R^2^_adj_
	***Right dlPFC GMV***					***Left dlPFC GMV***				
(Intercept)	0	(-0.24, 0.24)	0	1		0	(-0.20, 0.20)	0	1	
Sex	0.03	(-0.28, 0.34)	0.22	.828		0.26	(0.01, 0.52)	2.1	.044	
Age	-0.01	(-0.29, 0.27)	-0.09	.932		-0.11	(-0.34, 0.12)	-1.01	.322	
TIV	0.69	(0.4, 0.99)	4.81	< .001	.43^a^	0.91	(0.67, 1.15)	7.72	< .001	.64^a^
Involvement	0.07	(-0.19, 0.33)	0.54	.594		0.04	(-0.18, 0.25)	0.35	.726	
Positive Parenting	-0.17	(-0.46, 0.12)	-1.18	.247		-0.10	(-0.34, 0.14)	-0.87	.389	
Poor Monitoring	0.18	(-0.10, 0.46)	1.33	.193		0.07	(-0.16, 0.30)	0.65	.521	
Inconsistent Discipline	0.16	(-0.12, 0.43)	1.15	.258		0.05	(-0.18, 0.27)	0.40	.689	
Corporal Punishment	-0.07	(-0.36, 0.22)	-0.49	.628	.45^b^	0.1	(-0.13, 0.34)	0.91	.371	.63^b^
	***Right dlPFC CT***					***Left dlPFC CT***				
(Intercept)	0	(-0.27, 0.27)	0	1		0	(-0.26, 0.26)	0	1	
Sex	0.2	(-0.09, 0.49)	1.39	.174		0.21	(-0.07, 0.49)	1.55	.132	
Age	-0.34	(-0.65, -0.02)	-2.18	.037	.24^a^	-0.23	(-0.53, 0.07)	-1.57	.126	.21^a^
Involvement	0.26	(-0.02, 0.55)	1.86	.072		0.22	(-0.05, 0.49)	1.63	.113	
Positive Parenting	0	(-0.33, 0.32)	-0.02	.984		-0.05	(-0.36, 0.26)	-0.34	.739	
Poor Monitoring	-0.04	(-0.35, 0.27)	-0.25	.803		-0.15	(-0.45, 0.15)	-1.02	.315	
Inconsistent Discipline	-0.07	(-0.38, 0.24)	-0.49	.628		0.09	(-0.20, 0.39)	0.63	.532	
Corporal Punishment	-0.24	(-0.57, 0.08)	-1.53	.137	.30^b^	-0.44	(-0.75, -0.14)	-2.94	.006*	.37^b^
	***Right Hippocampus***					***Left Hippocampus***				
(Intercept)	0	(-0.23, 0.23)	0	1		0	(-0.25, 0.25)	0	1	
Sex	-0.11	(-0.4, 0.19)	-0.73	.47		-0.13	(-0.45, 0.19)	-0.83	.415	
Age	0.02	(-0.25, 0.29)	0.13	.895		-0.01	(-0.3, 0.28)	-0.07	.945	
TIV	0.64	(0.36, 0.92)	4.62	< .001	.48^a^	0.62	(0.31, 0.92)	4.13	< .001	.45^a^
Involvement	-0.07	(-0.32, 0.18)	-0.58	.565		-0.03	(-0.3, 0.24)	-0.25	.805	
Positive Parenting	0.28	(0, 0.56)	2.07	.047		0.13	(-0.17, 0.43)	0.88	.386	
Poor Monitoring	-0.17	(-0.44, 0.1)	-1.3	.204		-0.13	(-0.42, 0.16)	-0.93	.358	
Inconsistent Discipline	0.04	(-0.23, 0.31)	0.30	.763		0.07	(-0.22, 0.36)	0.50	.618	
Corporal Punishment	0.04	(-0.23, 0.32)	0.30	.764	.49^b^	-0.09	(-0.38, 0.21)	-0.58	.565	.40^b^
	***Right Amygdala***					***Left Amygdala***				
(Intercept)	0	(-0.2, 0.2)	0	1		0	(-0.21, 0.21)	0	1	
Sex	0.14	(-0.12, 0.39)	1.07	.293		-0.19	(-0.47, 0.08)	-1.42	.164	
Age	-0.02	(-0.26, 0.21)	-0.19	.854		-0.18	(-0.43, 0.07)	-1.46	.156	
TIV	0.71	(0.47, 0.96)	5.95	< .001	.46^a^	0.58	(0.32, 0.84)	4.55	< .001	.53^a^
Involvement	0.07	(-0.15, 0.28)	0.65	.517		0.07	(-0.16, 0.29)	0.58	.565	
Positive Parenting	0.49	(0.25, 0.73)	4.14	< .001^a^		0.32	(0.06, 0.57)	2.53	.017	
Poor Monitoring	-0.03	(-0.27, 0.2)	-0.29	.776		-0.03	(-0.28, 0.22)	-0.23	.817	
Inconsistent Discipline	0.02	(-0.22, 0.25)	0.14	.893		0.01	(-0.23, 0.26)	0.09	.93	
Corporal Punishment	0.16	(-0.08, 0.39)	1.32	.196	.62^b^	0.15	(-0.1, 0.4)	1.21	.237	.57^b^

***Note:*** GMV = grey matter volume; CT = cortical thickness; β = standardized beta; SE B = standard error for the unstandardized beta; R^2^_adj_ = adjusted R^2^ of ^a^models with control regressors only vs. ^b^full models; dlPFC = dorsolateral prefrontal cortex; TIV = total intracranial volume. *Significant on adjusted significance level of *p* < 0.006 (Bonferroni correction for multiple comparison).

### 3.4 Parenting behaviors and late adolescents’/young adults’ brain structure (aim 2.2)

None of the parenting behaviors collected during childhood showed a significant association with brain structure at age 22 years when including data of the full cohort 2 (Table S12). Based on significant findings on two ROIs in cohort 1 (right amygdala GMV and left dlPFC CT), follow-up analyses were conducted in a matched subgroup of cohort 2 (including similar levels of varying parenting behaviors, sex, age and parental education). The significance level was corrected for the two tests conducted (*p*/2 =.025). Re-evaluating the positive association between right amygdala volume and positive parenting identified in cohort 1 using parenting reports and neuroimaging data of the matched cohort 2 group, revealed a significant negative effect (*β* = -0.39, CI(95%) = [-0.63, -0.15], *t*(31) = -3.29, *p* = .002**;** Fig. 2**C**). The effect remained significant when parental education was included as a covariate (*β* = -0.36, CI(95%) = [-0.61, -0.11], *t*(30) = -2.95, *p* = .006). Corporal punishment was not significantly associated with left dlPFC thickness in the matched cohort of late adolescents/young adults (*β* < 0.01, CI(95%) = [-0.10, 0.10], *t*(32) = 0.01, *p* = .996). Variance inflation factors (all < 2) and correlation coefficients of all regressors are depicted in Table S13 and Figure S5.

### 3.5 Post-hoc analyses

#### 3.5.1 Age interaction

We examined whether age moderated the associations between parenting behaviors and brain structure in cohort 1, which covered a relatively wide developmental age range (7–14 years). For each parenting behavior that showed a significant association with brain structure in the primary analyses, we repeated linear regression models adding a parenting × age interaction term. Neither the interaction between positive parenting nor corporal punishment was significantly associated with right amygdala GMV or left dlPFC thickness, respectively (*β* = -0.06, CI(95%) = [-0.29, 0.17], *t*(30) = 0.50, *p* = .622; *β* = 0.08, CI(95%) = [-0.27, 0.43], *t*(31) = 0.45, *p* = .653).

#### 3.5.2 Sensitivity analyses

*Control-variables-based sensitivity analyses.* We conducted additional sensitivity analyses (besides parental education) to examine the robustness of the observed associations under alternative model specifications. All models showing significant effects were re-estimated multiple times with thematically grouped control variables added per model to limit the total number of predictors and reduce overfitting risk. The models accounted for a broad range of parental, child, family, and contextual factors (including parental mental health, household income, family structure, school experience, early developmental indicators, physical activity, and nutrition, depending on data availability for each cohort). Across both cohort 1 and the matched cohort 2 subgroup, the direction and magnitude of the associations were largely stable across models. Complete model specifications, variable characterizations, and results are reported in the Supplement (Supplementary Results, Figure S3, Table S11).

*Informant-related sensitivity analyses.* Because parent- and child-reported APQ measures differ in reference frame (i.e., referring to one vs. both caregivers) and a small proportion of child/adolescent-reported data (∼15%) was included in the matched-subgroup analyses, we repeated these analyses using only parent-reported parenting assessments (i.e., the latest parent-reported time point at age 11). Results were highly similar to the primary analyses: positive parenting remained significantly negatively associated with right amygdala volume, whereas corporal punishment was not associated with left dlPFC cortical thickness (see Supplement).

## 4 Discussion

By analyzing two developmental community cohorts, a cross-sectional study and a large longitudinal prospective cohort study (*N* > 1000) spanning over 15 years, with both behavioral reports and neuroimaging data, we demonstrate the following key outcomes: Behaviorally, both parents and children/adolescents reported a lower use of adaptive and maladaptive parenting practices over time, indicated by a general decrease in behaviors reported with increasing age of the children (Lansford et al., 2021). Consistent with previous studies (Frick et al., 1999, Florean et al., 2022), parents’ and children’s reports of parenting behaviors were positively correlated, suggesting that both informants have a similar perspective on parenting behaviors when asked individually. Neurally, we demonstrate that parenting behaviors are related to emotion regulatory brain structures in childhood and late adolescence/young adulthood, with the direction and presence of these associations differing as a function of developmental stage and cohort composition. More specifically, positive parenting was associated with larger amygdala volume during childhood. Positive parenting behaviors during childhood were linked to smaller amygdala volume in a matched subgroup of late adolescents/young adults with a socioeconomic background and group composition comparable to the childhood cohort tested. Furthermore, corporal punishment was negatively linked to left dlPFC thickness in childhood. Notably, these parenting behaviors-brain associations were robust across sensitivity analyses controlling for parental mental health and child general and mental health.

### 4.1 Parenting behaviors across development: age-related changes and informant perspectives

A key strength of the present analyses is the opportunity to examine longitudinal changes in normative-range parenting behaviors across childhood and adolescence in a community sample, spanning a period of 10 years (cohort 2). Overall, both adaptive parenting behaviors (i.e., involvement and positive parenting) and maladaptive parenting behaviors (i.e., inconsistent discipline and corporal punishment) decreased over childhood and adolescence (7–17 years). This decline aligns with cross-sectional (Frick et al., 1999, Shelton et al., 1996, Florean et al., 2022) and longitudinal evidence with similar measures of parenting behaviors (i.e., behavioral control measured by the Parental Acceptance-Rejection/Control Questionnaire (Lansford et al., 2021, Rohner and Khaleque, 2005); corporal punishment (Gard et al., 2022) measured by items of the physical aggression scale of the Parent–Child Conflict Tactics Scale (Straus et al., 1998)). Our observation of increasing poor monitoring scores over time may reflect an age-appropriate reduction in parental monitoring behaviors (Lionetti et al., 2019). Parent-dependence is thus shifting to adolescent independence. Importantly, while such reductions in parental monitoring may be developmentally normative, insufficient monitoring during sensitive developmental periods has also been linked to increased risk-taking and difficulties in emotion regulation, highlighting the importance of considering developmental timing and contextual factors when interpreting these trajectories (Dittus et al., 2023, Balan et al., 2017). Conversely, inconsistent discipline did not follow a consistent decline, remaining more stable than other behaviors. Stability in inconsistent discipline may be problematic, as it reflects a lack of predictability in the environment, which is a predictor of internalizing (Balan et al., 2017), externalizing and emotional regulation problems throughout childhood and adolescence (Duncombe et al., 2012). Such sustained environmental unpredictability may place ongoing demands on developing emotion regulation systems, even in the absence of detectable structural brain differences in the present study. The observed changes in parental practices underscore the importance of considering children’s age when interpreting parenting behaviors across different stages of individuals’ development (Michael et al., 2024).

Studies support the notion that parenting behaviors change as children enter puberty (Lansford et al., 2021), paralleling a time window when substantial biological (e.g., neural, hormonal), social and behavioral changes occur (Blakemore et al., 2010, Dahl and Gunnar, 2009). For instance, children entering puberty might experience altered emotional responses and heightened frequency of mood swings (Blakemore et al., 2010, Dahl and Gunnar, 2009). They seek greater independence of their parents, which may in turn affect parental behaviors (e.g., communication (Keijsers and Poulin, 2013) or co-regulation of emotions (Lougheed et al., 2016)), potentially contributing to non-uniform developmental patterns in parenting over time. Evidence of U-shaped or inverted U-shaped trajectories has been reported for parenting constructs not tested here (e.g., parental warmth, rule-setting, solicitation (Lansford et al., 2021; Keijsers et al., 2009), which seem to be age- or puberty-dependent. Our results do not support an (inverted) U-shaped pattern for the specific parenting behaviors studied, noting that the gap between ages 11 and 14 limits sensitivity to early-adolescent peaks. Nonetheless, the observed patterns, characterized by overall decreases across childhood and adolescence with periods of relative stability (e.g., ages 8–9), are consistent with the idea that parents adapt their strategies in response to specific developmental milestones (e.g., school transitions), rather than following a strictly linear decline. Furthermore, while we observed group-level trends, individual variability in parenting trajectories is likely (see, for example (Gard et al., 2022), highlighting the need for future studies to examine interindividual differences in parenting development and their implications for emotion socialization and associated brain structures.

Comparing parental and children’s reports on parenting behaviors at age 11 years revealed a positive relation, indicating similar experiences by parents and children. Our findings align with some, but not all previous studies (Shelton et al., 1996). For example, Shelton et al. (1996) found significant correlations in reports of adaptive parenting behaviors (i.e., involvement and positive parenting) and corporal punishment, but no correlation in reports of poor monitoring and inconsistent discipline between ages 6 and 13. Florean et al. (2022) reported significant differences in poor monitoring reports from ages 10–18, while all other parenting behaviors did not differ between parental and child report. Another study noted similarity in reports of all APQ parenting behaviors, but this similarity varied with age (Russell et al., 2016). Interestingly, 12-year-olds align less with parents’ reports of involvement and positive parenting than 15-to-16-year-olds, but more closely than 9-year-olds (Russell et al., 2016). Overall, correlation values were modest, which may partly reflect differences in reference frame between parent- and child-reported parenting measures.

### 4.2 Parenting behaviors and brain structure: associations in childhood and late adolescence

In line with past research, we report that positive parenting behaviors are associated with GMV of limbic brain structures during childhood (Whittle et al., 2014). Although we initially hypothesized smaller amygdala volume in association with adaptive parenting during childhood, our findings indicate a positive association at this developmental stage. Specifically, we identified that a higher reported use of positive parenting behaviors is associated with significantly larger amygdala GMV in children (cohort 1; age range: 7–14 years). It has been suggested that parenting behaviors impact structural brain correlates associated with emotion socialization and emotion regulation skills, which reflect children’s ability to cope appropriately with emotions (Tan et al., 2020). Previous evidence has similarly linked positive parenting behaviors with larger amygdala volumes in older children (12-year-olds (Whittle et al., 2014)), however, an association to smaller amygdala volumes have been reported in younger children (6-year-olds (Lee et al., 2019) and 10-year-olds (Bernier et al., 2019)). Differences in the direction of associations may be due to the age range of the children, differences in the type of parenting behavior reported or based on the type of report used (prospective or concurrent to neuroimaging measures obtained) (Tottenham and Sheridan, 2010). For example, Lee et al. (2017) and Bernier et al. (2019), reporting a negative association between adaptive parenting behaviors and amygdala volume, assessed maternal sensitivity behaviors during infancy, while neuroimaging was measured in early/middle childhood. Contrarily, studies reporting a positive association between positive parenting and amygdala volume (Whittle et al., 2014) (including the current study) measured positive parenting at the same age as amygdala volumes in middle childhood/early adolescence.

A potential neurobiological mechanism might explain why positive parenting was associated with larger amygdala volume in the current study. This positive association may reflect effects of positive parental reinforcement behaviors on children’s biology. The items of the positive parenting scale (Frick, 1991) used here capture positive reinforcement behaviors, such as how often parents reward, compliment, kiss or hug their child in response to good behavior. While early research predominantly linked the amygdala with negative emotions and fear learning (LeDoux, 2003), more recent studies in mammals and humans have also associated the amygdala with positive emotions and positive reinforcement learning (Bonnet et al., 2015, Murray, 2007, Namburi et al., 2015). However, direct evidence linking reinforcement-related experiences to structural amygdala development remains limited. Notably, a separate line of research has associated larger amygdala volume with early life adversity and maladaptive socioemotional outcomes (Tottenham et al., 2010). Crucially, our findings do not necessarily contradict prior reports linking larger amygdala volume in childhood to trauma or less desirable outcomes, as developmental timing, environmental context, and longitudinal trajectories of amygdala maturation likely determine whether larger volume reflects adaptive plasticity or accelerated stress-related development.

In line with our hypothesis, reports of early positive parenting behaviors were significantly associated with smaller amygdala volume in late adolescents/young adults (around age 22; cohort 2) within a subgroup matched on parental education (as a proxy for socioeconomic background) and composite parenting behavior scores. To the best of our knowledge, the effects of early adaptive parenting behaviors on brain structure in late adolescence/young adulthood have rarely been reported, in contrast to a larger body of work linking maladaptive parenting behaviors or early adversity to later neural outcomes. Studies focusing on maladaptive parenting behaviors or adverse childhood experiences have reported larger amygdala volumes in late adolescence (Mehta et al., 2009) and young adulthood (Pechtel et al., 2014, Evans et al., 2016). Importantly, amygdala volume follows a normative non-linear growth trajectory, often described in the form of an inverted U-shaped curve across childhood and adolescence, peaking around late childhood (Uematsu et al., 2012) to late adolescence (Wierenga et al., 2014, Narvacan et al., 2017, Herting et al., 2018) depending on the study design, age range or model tested. This normative peak may be influenced by both positive and negative environmental factors, including maladaptive or adaptive parenting behaviors (Tottenham and Sheridan, 2010, Kahhalé et al., 2023). In line with the associations found here, larger amygdala volume before or around this peak may reflect normative development, whereas enlarged amygdala volumes persisting into young adulthood may indicate delayed maturation (Uematsu et al., 2012, Callaghan and Tottenham, 2016). Repeated measures neuroimaging studies spanning the amygdala’s peak growth period are therefore needed to more directly test these developmental hypotheses. Rather than viewing amygdala volume as a uniform marker of stress or pathology, our results support the interpretation that larger volume in childhood, when considered alongside normative pruning by young adulthood, may reflect a developmentally appropriate neural scaffold supporting emotional development.

Notably, we did not find an association between positive parenting and amygdala volume in the full group of late adolescents/young adults. This discrepancy may be partly explained by differences in parental education, which was on average lower in the full adolescents/adults group compared to both the childhood cohort and the matched subgroup. Parental education represents one dimension of socioeconomic background, which can be linked to parenting behaviors and developmental outcomes (Callahan and Eyberg, 2010, Conger et al., 2010). The interplay between socioeconomic disadvantage, increased stress exposure, and reduced opportunities for adaptive parenting may influence corticolimbic development (e.g., poverty-related deceleration of amygdala development (Qiu et al., 2025; Baysarowich et al., 2025) and associated behavioral outcomes.

Consistent with our hypothesis, corporal punishment was significantly negatively associated with dorsolateral prefrontal cortical thickness in children. Our findings align with research showing that adverse childhood experiences, such as corporal punishment, are associated with reduced prefrontal cortical thickness in children and adolescents (Gold et al., 2016, Kelly et al., 2013), as well as altered prefrontal cortical thickness development in adolescents (Whittle et al., 2016). The prefrontal cortex plays a critical role in the acquisition of emotion regulation skills (Ochsner et al., 2012). Moreover, alterations in prefrontal structure and function have been associated with increased vulnerability to psychopathology (Zilverstand et al., 2017). Furthermore, altered prefrontal cortical thickness and maturation have been linked to reduced cognitive and behavioral functioning in children and late adolescents (Whittle et al., 2016, Ronan et al., 2020), suggesting a potential mediating pathway between maladaptive parenting and later outcomes (Whittle et al., 2016). While the present study focused on parenting-brain associations, future research integrating mental health outcomes is needed to test such models more directly. Importantly, parenting interventions have been shown to reduce maladaptive parenting behaviors, including corporal punishment, and to support children’s healthy development (Feinfield and Baker, 2004, Grogan-Kaylor et al., 2019, Lochman and Wells, 2002). In fact, parenting interventions can be as effective for as cognitive behavioral therapy for treating childhood anxiety disorders (Lebowitz et al., 2019). By reducing exposure to maladaptive parenting practices, such interventions may therefore also mitigate adverse effects on brain development and support long-term emotional well-being.

### 4.3 Limitations

Several limitations should be noted. First, childhood and late adolescence/young adulthood parenting-brain associations were not measured within the same cohorts, precluding direct inferences about within-person developmental trajectories. Future research may further include neuroimaging measures at several time points within the same cohort. Second, participants in the late adolescents/young adults neuroimaging group were recruited as part of a study on victimization, which may partly explain differences in parental education and other group characteristics between cohorts. We aimed to reduce this bias by matching cohorts based on parental education, among other variables. While parenting-brain associations remained significant after controlling for parental education, residual group differences cannot be fully excluded. Third, in line with the majority of existing literature (Buss et al., 2007, Edmiston et al., 2011, Schneider et al., 2012, Stepanous et al., 2023, Narita et al., 2010, Kim et al., 2010, Yang et al., 2018, Kahhalé et al., 2023), parenting behaviors were based on questionnaire reports and predominantly maternal reports (Buss et al., 2007, Schneider et al., 2012, Kim et al., 2010) were used here and only few paternal behaviors were reported. Fourth, small sample sizes and consequently power considerations, especially in our childhood neuroimaging group warrant caution in the interpretation of the current findings. Fifth, information on participants’ ethnicity was not available in the child cohort (cohort 1). Another limitation concerns differences in reference frame between informants: parent-reported parenting ratings primarily reflect the behavior of the reporting parent, whereas child and adolescent reports refer to parenting practices of both parents, which may limit direct comparability across informants. Sixth, we had a relatively wide age range (7–14 years) in our child neuroimaging group (cohort 1). Given the limited sample size in cohort 1, statistical power was insufficient to examine more fine-grained age-by-parenting interaction effects beyond exploratory moderation analyses conducted for the significant associations. Finally, while we accounted for a broad set of alternative parental, child, family, and contextual variables in sensitivity analyses, we cannot rule out the influence of additional variables, such as sleep or neighborhood characteristics, which have been linked to brain development in prior work (Jalbrzikowski et al., 2021, Hackman et al., 2021).

## 5 Conclusion

The current study provides longitudinal evidence that parenting behaviors change over time, with both adaptive and maladaptive behaviors decreasing as children age, while poor monitoring tends to increase. Importantly, children’s and parents’ reports on parenting behaviors showed positive alignment. Extending these behavioral findings to neurodevelopment, the current study identified distinct associations between early parenting behaviors and corticolimbic brain structures in childhood and late adolescence/young adulthood. Specifically, positive parenting was associated with amygdala volume in children and in a matched subgroup of late adolescents/young adults, whereas corporal punishment was negatively associated with dorsolateral prefrontal cortical thickness in children. These findings highlight the importance of considering developmental timing and contextual factors when interpreting parenting-brain associations. Parenting programs, such as the Triple P-Positive Parenting Program (Sanders et al., 2014), may provide an effective avenue to promote adaptive parenting behaviors and reduce maladaptive practices, thereby supporting children’s long-term behavioral and neurodevelopmental health.

## CRediT authorship contribution statement

**Mirjam Habegger:** Writing – review & editing, Writing – original draft, Visualization, Validation, Project administration, Methodology, Investigation, Formal analysis, Conceptualization. **Plamina Dimanova:** Writing – review & editing, Methodology, Investigation, Formal analysis, Conceptualization. **Réka Borbás:** Writing – review & editing, Visualization, Methodology, Investigation, Formal analysis, Conceptualization. **Elena Federici:** Writing – review & editing, Investigation. **Denis Ribeaud:** Writing – review & editing, Resources, Project administration, Investigation, Funding acquisition, Data curation. **Manuel P. Eisner:** Writing – review & editing, Validation, Resources, Project administration, Investigation, Funding acquisition, Data curation. **Todd A. Hare:** Writing – review & editing, Validation, Supervision, Resources, Methodology, Investigation, Funding acquisition, Data curation, Conceptualization. **Nora Maria Raschle:** Writing – review & editing, Visualization, Validation, Supervision, Resources, Investigation, Funding acquisition, Data curation, Conceptualization.

## Funding

This study was funded by the Hochschulmedizin Zürich (NMR, TAH). NMR receives further funding from 10.13039/501100001711Swiss National Science Foundation (Grant No. 105314_207624), the University of Zurich Research Priority Program “Adaptive Brain Circuits in Development and Learning,” and the Jacobs Foundation CRISP program. Furthermore, funding from the 10.13039/501100001711Swiss National Science Foundation (Grants 405240–69025, 100013_116829, 100014_132124, 100014_149979, 10FI14_170409), the 10.13039/501100003986Jacobs Foundation (Grants 2010–888, 2013–1081–1), the Jacobs Center for Productive Youth Development, the Swiss Federal Office of Public Health (Grants 2.001391, 8.000665), the Canton of Zurich’s Department of Education, the Swiss Federal Commission on Migration (Grants 03–901 (IMES), E-05–1076), the Julius Baer Foundation, and the Visana Foundation is gratefully acknowledged (DR, MPE).

## Declaration of Competing Interest

All authors declare that they have no known competing financial interests or personal relationships that could have appeared to influence the work reported in this paper.

## Data Availability

Data will be made available on request.

## Footnotes

Appendix A Supplementary data associated with this article can be found in the online version at doi:10.1016/j.dcn.2026.101728.
