# Supplementary material for: Parenting behaviors over time and their effects on cortical and limbic brain structure in children and young adults
Source: Dev Cogn Neurosci. 2026 Apr 19;79:101728. doi: 10.1016/j.dcn.2026.101728 (PMC13129451; doi:10.1016/j.dcn.2026.101728)
Supplement: Table S1 — Supplementary material [file mmc1.docx]

# Supplementary Material

**Supplementary Methods.** Participants and group demographics, socioeconomic status, parenting behaviors, structural image acquisition and preprocessing, quality control, data analysis and matched subgroup approach.

**Table S1*.*** Cohort 2: Descriptive values of behavioral data across all waves

**Table S2*.*** Study cohorts and available data at each assessment wave (W)

**Table S3*.*** Parental education: transformation of values between cohorts

**Table S4*.*** Parenting behaviors: Reliabilities/ internal consistency – cohort 1

**Table S5*.*** Parenting behaviors: Reliabilities/ internal consistency – cohort 2

**Table S6*.*** Parenting questionnaire’s instructions and items for adolescents’ reports (age 11 to 17, cohort 1)

**Table S7*.*** Descriptives of composite parenting scores across all neuroimaging (sub)groups

**Figure S1.** Representative MCMC trace plots for imputation diagnostics (Involvement subscale, W1**-**W4).

**Figure S2.** Density overlays of observed and imputed data.

**Table S8.** Stability and reliability of parenting behaviors across time points (7-11 years) in the neuroimaging group of cohort 2

**Table S9.** Significant regressions of cohort 1 with parental education added as a proxy for SES

**Table S10.** Variance inflation factors (VIF) of cohort 1 regressors

**Figure S3.** Multiple sensitivity analyses for all significant parenting behavior-brain associations.

**Table S11.** Multiple sensitivity analyses for all significant parenting behavior-brain associations

**Figure S4.** Spearman correlation coefficients between all regressors of cohort 1 models.

**Table S12.** Regressions of parenting behaviors on brain GMV and dlPFC CT in late adolescents/young adults.

**Table S13.** VIF of cohort 2 regressors

**Figure S5.** Spearman correlation coefficients between all regressors of cohort 2 models.

This supplementary material has been provided by the authors to give readers additional information about their work.

# Methods

## Participants and group demographics

**Cohort 2.** Participants were initially selected with a cluster stratified randomized sampling procedure. At the beginning of the study in 2004, 1675 children from 56 public schools were randomly selected. School sizes and socio-economic backgrounds of school districts were taken into account for the stratification approach. At baseline, 1360 out of 1675 children participated. The sample was representative of children attending first grade at public schools in Zurich. Re-recruitment resulted in an increased participation to 1,446 children/adolescents at wave 5 (age 15 years) in 2013^1^.

**Neuroimaging data, cohort 2.** A subsample (*n* = 200) of cohort 2 were invited at age 21/22 years to complete neuroimaging, in addition to further assessments including stress response measures and DNA sequencing not relevant to the present paper. For 135 participants both structural MRI and APQ data was available. One transgender individual taking hormones needed to be excluded, thus data from 134 participants were used for the current study. The MRI subsample was initially selected to analyze effects of victimization and bullying.

### Parental Education

Parental education was used as a proxy for socioeconomic status (SES). To ensure comparability across cohorts, maternal and paternal ISCED scores in cohort 1 were transformed to match the parental education scoring used in cohort 2 (see Table S3). Scores were averaged across parents to obtain a composite parental education score per child. Mean parental education was included as covariate in relevant models as described in the main article.

**Neuroimaging subgroup of cohort 2:** In the MRI subgroup two mothers and two fathers (from different families) did not complete compulsory school, indicating below average SES from at least one of the parents for four children in this subgroup. Mothers had an average education level of 6.43 (median = 6, SD = 2.95, range from 1 = compulsory school not completed to 10 = University degree), while fathers average education level was 7.20 (median = 9, SD = 2.99, range from 1 to 10). When averaging these education scores of both parents one participant had a mean parental education score below two (2 = compulsory school completed), indicating a very low socio-economic status. Maximum education level was both parents having completed University. As in the full group 2, parental yearly income ranged from below 19’200 USD to more than 144’000 USD (in 2004).

## Parenting behaviors

Parenting behaviors were assessed using the German version of the Alabama Parenting Questionnaire (APQ^2,3^). The APQ includes 35 5-point Likert-type items: 1 = “never”, 2 = “almost never”, 3 = “sometimes”, 4 = “often”, 5 = “always”^3^, which are used to build the two adaptive (involvement and positive parenting) and the three maladaptive (poor monitoring, inconsistent discipline, and corporal punishment) parenting scales. The five subscales of the APQ have demonstrated adequate to good reliability in terms of internal consistency (ranging from α = .67–.80) except for corporal punishment (α = .46) in 6-13 year-olds^4^. Minor adaptations (**Table S4**) from the 35 original items in cohort 2 for parents of children between 7-11 years of age and adolescents from 13-17 years of age were made to assure age-appropriateness in wording for Swiss German speaking youth, also addressing ethical considerations. Further, for adolescents’ reports, the highest two APQ answer categories were combined into one category “often”, thus items were based on a 4-point Likert-type scale: 1 = “never”, 2 = “almost never”, 3 = “sometimes”, 4 = “often”. Reliabilities of all parenting scores can be seen in Tables S5-S6.

For parent-child alignment analyses at age 11, parental and child ratings were compared using only the items available from both informants. Resulting parenting scores showed low internal consistencies for both parental (involvement: α = .39, positive parenting: α = .12, poor monitoring: α = .59, inconsistent discipline: α = .32 and corporal punishment: α = .59) and children’s scores (involvement: α = .44, positive parenting : α = .37, poor monitoring: α = .46, inconsistent discipline: α = .32 and corporal punishment: α = .51). **These reduced-item scores were used exclusively for the parent-child alignment analysis and were not used in longitudinal mixed-effects models or parenting-brain analyses**.

## Structural image acquisition and pre-processing

### Image acquisition

Structural T1-weighted MPRAGE data was acquired on a Siemens 3T Prisma MR scanner using 20-channels head coil (cohort 1) and on a Philips Achieva 3T scanner using a 8-channels head coil (cohort 2). Cohort 1, specifics: voxel size: 1.0×1.0×1.0 mm; TR=1900ms; TE=3.42ms; TA=4.26; flip angle=9 degrees; field of view=256x256mm, 192 slices with a slice thickness of 1.00mm. Cohort 2, specifics: voxel size: 1.0×1.0×1.0 mm; TR=8.3ms; TE=3.9ms; TA=357.77; flip angle=8 degrees; field of view=256x256mm, 181 slices with a slice thickness of 1.00 mm.

### Image preprocessing

Structural MRI data of both groups was preprocessed with FreeSurfer (v7.1.0 for cohort 1 and v7.2.0 for cohort 2; <https://surfer.nmr.mgh.harvard.edu/>) using the automated ‘recon-all’ stream (motion correction, intensity-normalization, Talairach-registration, stripping of skull, removal of non-brain tissue, segmentation, tessellation, smoothing, and parcellation of the cortex^5,6^). GMV of limbic regions (i.e., the bilateral amygdala and hippocampus) derived from automatic segmentation, whereas GMV and CT of the dlPFC (i.e., caudal and rostral middle frontal gyrus) was reckoned as defined in the Desikan/Killiany atlas^7^. Bilateral middle frontal gyrus regions were used based on findings of previous literature on parenting and emotion regulation brain network^8–10^. To obtain one GMV and one CT measure for each left and right dlPFC, GMV values of caudal and rostral middle frontal regions^7^ were summed and CT values averaged, separately for both hemispheres. Additionally, total intracranial volume (TIV) was extracted to include as a covariate in the analyses later.

**Neuroimaging quality control: comparison between cohorts**

For younger participants (cohort 1), age-appropriate analyses protocols were performed^11,12^, including quality control of cortical gray matter segmentation through visual inspection and manual correction. Following preprocessing of structural data in FreeSurfer v7.1.0 (<https://surfer.nmr.mgh.harvard.edu/>) using the standard "recon-all" pipeline, we visually inspected the quality of segmented and reconstructed images. If inadequate quality was identified, such as inaccuracies in the delineation of the pial surface (separating cerebrospinal fluid and gray matter), manual corrections were conducted. Subsequently, the preprocessing steps post-segmentation were repeated. This process of visual inspection, manual correction, and preprocessing was iterated for each participant until no further edits were necessary for the delineation of the pial and white surfaces.

To ensure comparability between cohort 1 and cohort 2, the same rater repeated this quality control procedure for 10 late adolescents/young adults from cohort 2, who represent the whole spectrum of T1 quality in our regions of interest, including participants with poor, medium, and very good quality in those regions. After quality control and manual correction, all GMV values of the Desikan/Killiany atlas^7^ were extracted and compared between manually corrected and uncorrected values for the 10 trial participants of cohort 2. Correlation coefficients within all cortical regions were very high (M = 0.986, *SD* = 0.033). For regions of interest, correlations were *r* = 0.998 for left middle frontal gyrus and *r* = 0.998 for right middle frontal gyrus. T-tests to compare mean differences were non-significant (right middle frontal gyrus: M_1_ = 24’932, M_2_ = 24’939.9, t = -0.01, *p* = .995; left middle frontal gyrus: M_1_ = 24’952, M_2_ = 25’011.8, t = -0.04, *p* = .966). Subcortical regions were not manually corrected or compared. Since the applied manual corrections did not lead to significant enhancement of data quality in the adult group 2, manual corrections were not further applied to the cohort 2 sample. Analyses were conducted with the original uncorrected GMV values as well as the uncorrected cortical thickness values.

## Data analyses

All data analyses were run with R^13^ in RStudio^14^.

In case of missing APQ items, the respective subscale was built by averaging all other available items (per person) belonging to this subscale. Thus, a subscale score (e.g., involvement) for a person was only missing if all items of this subscale were missing.

Cohort 2: repeated measures data. For cohort 2 longitudinal analyses, missing data was imputed based on the available repeated measures assessments. Specifically, if an APQ subscale score was missing for a person at a given time point, data of this time point was imputed using multivariate predictive mean matching with the ‘mice’ package in R (m = 30 datasets with 5 Markov Chain Monte Carlo (MCMC) iterations)^15^ based on other time points score of all parenting variables and the child’s sex. To ensure stable and parsimonious models, the ‘quickpred’ function was used to select predictors for each target variable based on a minimum correlation of r = 0.10 and a minimum usable case proportion of 0.25. Imputation quality was verified through visual inspection of MCMC trace plots and density overlays (see Figures S1 and S2). The trace plots demonstrated excellent convergence and mixing of the chains, while the density overlays confirmed that the distribution of imputed values remained highly consistent with the observed data, indicating that the original data structures were well-preserved. For information about the amount of data missing at each time point, see **Table S2**.

### Matched subgroup approach: Matching process

Participants of cohort 1 and neuroimaging cohort 2 were matched based on the following variables: adaptive parenting, maladaptive parenting, age, sex, and parental education. Before matching, for both cohorts, the two adaptive subscales (involvement and positive parenting) and the three maladaptive subscales (poor monitoring, inconsistent discipline, and corporal punishment) were averaged at each assessment wave to receive composite adaptive and maladaptive parenting behavior scores, respectively. The matching was then conducted by using the ‘find.matches’ function of the ‘Hmisc’ R-package^16^ in combination with manual selection. To obtain suitable matches, tolerance thresholds (i.e., maximum allowed differences between cohorts) were iteratively adjusted across matching variables over several rounds until a match was identified for each participant in cohort 1. For age, the maximum tolerated difference between matched participants was 2 years. Consequently, for ~85% of matches, parenting scores in cohort 2 were taken from the assessment wave closest in age to the cohort 1 assessment; in the remaining of cases, the second-closest assessment wave was used. Maximum differences between the final groups on each variable were as followed: sex = no difference, age = 2 years, parental education = 2.5 units (scale: 1-10), composite adaptive and composite maladaptive parenting = 0.45 units (scale: 1-5). **Table S5** depicts mean values and standard deviations of composite parenting scores of both matched cohorts.

# Results

**Sensitivity analyses**

***Control-variables-based sensitivity analyses.*** To assess the robustness of the primary parenting behavior-brain associations, we conducted a series of control-variables-based sensitivity analyses in which thematically related covariates were added jointly to the main models in separate specifications. This approach allowed us to evaluate potential confounding across multiple domains while limiting model complexity and preserving sample size. Detailed descriptions of all measures included in these sensitivity analyses are provided below. Results can be seen in Figure S3 and Table S11.

**Cohort 1**

Household income:

- Besides parental education, we specified an additional model including household income as a regressor. Gross yearly household income was assessed as an ordered categorical variable with 7 response options ranging from below CHF 4’500 to CHF 89’000 or more.

Maternal (mental) health:

- Maternal age
- Maternal mental health problems were assessed using the Brief Symptom Inventory (BSI)^17^. The BSI is used to assess subjective physical and psychological symptoms. A global severity index was calculated across all 53 items representing an aggregate across the following problem scales: somatization, obsession-compulsion, interpersonal sensitivity, depression, anxiety, hostility, phobic anxiety, paranoid ideation psychoticism.
- **Parental stress** was assessed using the **Parental Stress Scale** (PSS^18^). A total score across 18 items rated on a 5-point Likert scale, reflecting stress related to the parenting role, was used.
- **Emotion regulation strategies** were assessed using the Emotion Regulation Questionnaire (ERQ^19^), a questionnaire consisting of 10 items assessing the habitual use of cognitive reappraisal and expressive suppression.

Child socioemotional skills and mental health problems:

- Socioemotional skills were assessed using the Inventory for the Assessment of Social Competence (IDS^20^). Total scores of the following tasked-based IDS subscales were used: **Emotion Recognition (**ability to perceive and identify one’s own and others’ emotions), **Emotion Regulation (a**bility to regulate and manage emotional responses), **Social Behavior (adaptive** social behavior in interpersonal situations) and **Social Cognition (s**ocial-cognitive skills, such as perspective-taking and understanding social situations).
- Prosocial behavior was additionally assessed using the Prosocial subscale of the Strengths and Difficulties Questionnaire (SDQ^21^). The SDQ is a 25-items behavioral screening questionnaire filled out by parents to assess psychological adjustment of children and adolescents.
- Child mental health problems were assessed with the Child Behavior Checklist (CBCL/6-18^22^). The CBCL/6-18 is a parent-rated questionnaire to assess children’s behavioral and emotional problems with 113 items reported on a 3-point Likert scale (“not true”, “somewhat or sometimes true” and “very true or often true”). Broadband internalizing and externalizing problems scales were used which are aggregated from the anxious/depressed, withdrawn/depressed, somatic complaints (internalizing) and rule breaking behavior and aggressive behavior (externalizing). Sex- and age-normed T-scores were used.

Early motor and language development:

- **Early developmental milestones.** Age at onset of independent walking and age at first spoken word were separarately included in a regression model and coded as binary dummy variables, distinguishing children who reached the milestone between **6-12 months** versus **1-1.5 years**.

**Matched group of cohort 2**

Measures are further described in the cohort 2 (*z-proso* Project’s) handbooks^23,24^.

Maternal mental health and skills were all assessed at child age 8 years:

- Maternal depression in the last few weeks were measured with a slightly adapted version of the General Health Questionnaire (GHQ-12)^25^. The GHQ total score was used, which is the mean of 12 items answered on a 4-point Likert scale (from “better than usual”/”not at all” to “much less than usual”/”much more than usual”).
- Maternal self-control was assessed using a modified version of the Grasmick self-control scale based on Gottfredson and Hirschi’s General Theory of Crime^26^. The final scale comprised 15 items rated on a 5-point Likert scale, yielding subscales for impulsivity, risk seeking, short temper, self-centeredness, and guilt/shame. The total self-control score, an average across all 15 items, was used in the sensitivity analyses.
- Social desirability was measured using a z-proso-developed exploratory scale (12 yes/no items), of which four items identified via factor analysis were combined to form a social desirability score. Items referred to positively framed statements about child behavior and were answered using a binary yes/no response format.

Child socioemotional skills and mental health problems:

- Child externalizing problems, internalizing problems (anxiety/depression), and prosocial behaviour were assessed using the parent-reported Social Behaviour Questionnaire (SBQ)^27^. For each variable, we computed a composite score by averaging wave-specific SBQ scales across available assessments waves (W1, W3, and W4 for externalising and anxiety/depression; W1-W4 for prosocial behavior).

Family context:

- Interparental conflict related to childrearing was assessed at child age 8 years using the Parent Problem Checklist (PPCL)^28^. The PPCL comprises 16 items capturing disagreement over rules and discipline, open conflict, and the extent to which parents undermine each other in family management. Items were rated on a 5-point Likert scale (from not a problem to a very big problem). A total score computed by averaging across items was used in the current study.
- Harmony between parents was assessed using the abbreviated Dyadic Adjustment Scale (DAS-7)^29^. The scale includes items assessing relationship satisfaction, consensus, and cohesion, rated on 6- or 7-point Likert scales depending on the subscale. A total dyadic adjustment score was computed and included as regressor in the sensitivity regression models.
- Family climate was assessed at child age 8 years using a brief three-item scale measuring overall family cohesion and conflict^30^. Items were rated on a 5-point Likert scale (from very much disagree to very much agree). A composite family climate score was computed by averaging across items and used in the current analyses.
- Number of siblings at the baseline assessment (child age 7 years) was included as an ordinal variable.

School experience:

- School liking was measured with a single item (“How much do you like going to school?”) on a 4-point Likert scale ranging from not at all (0) to very much (3) at child age 8 years.
- Three school-related variables based on Ribeaud and Eisner^31^ were included:
  - **Cumulative school risk:** Multiple child- and teacher-reported school-related risk indicators (bullying, school problems, class cohesion, class changes and the teacher-child relationship) were combined, with the score calculated as the mean of available indicators multiplied by the total number of indicators.
  - **Teacher-reported school problems:** To provide a conservative specification alongside cumulative school risk, teacher-reported school problems at child age 8 years were included. School problems were assessed using the School Problems Checklist (7 items, e.g., “purposeful destruction of school materials/vandalism”).
  - **Violent peers:** Violent peers were assessed via child report at age 8 years, capturing whether the child’s friends engage in violent or delinquent behaviours.

Prenatal/perinatal exposures and child injury history:

- **Severe complications during pregnancy or birth** were included as a binary indicator (yes/no).
- **Maternal alcohol consumption during pregnancy** was included as a binary indicator (yes/no).
- **Child injury history** was assessed as the number of times the child had been injured since birth requiring a doctor visit (excluding injuries related to insect bites or allergic reactions) up until baseline assessment (age 7 years).

Physical activity and nutrition:

The following items from the z-proso leisure activities inventory^31^ were used:

- Participation in organised sport activities outside school (e.g., football, tennis, swimming) was assessed by parent report. Parents indicated how many days per week the child participated in organised sports (0-7 days).
- Healthy eating behaviour was indexed using an item assessing how many days per week the child ate an apple or other fruits or vegetables between meals (0-7 days).

***Informant-related sensitivity analyses.*** To further address potential limitations in informant comparability, we repeated the matched-cohort analyses with parent-reported parenting behaviors only. In this matched group, six child-reported observations from age 14 were used (15% of participants). For the sensitivity analyses, we used the assessment wave corresponding to the latest parent-reported parenting measure (age 11) for these participants. Results remained highly similar. Positive parenting continued to show a significant negative association with right amygdala volume (*β* =-.69, CI(95%)=[−1.10, −0.27], *t*(30)=-3.35, *p*=.002). Corporal punishment remained non-significantly associated with left dorsolateral prefrontal cortex (dlPFC) cortical thickness (CT) (*β*=.10, CI(95%)=[-0.29,.49], *t*(31)=-2.52, *p*=.606). Because variance inflation factors (VIF) indicated potential multicollinearity between positive parenting and involvement, we repeated the analysis excluding involvement. VIF values were then within acceptable limits (< 2) and the association between positive parenting and right amygdala volume remained significant (*β*=-.31, CI(95%)=[-0.55,-.06], *t*(31)=-2.55, *p*=.002).

**Table S1*.*** Cohort 2: Descriptive values of behavioral data across all waves

**A)**

|  | W1: N = 1247 | | |  | W2: N = 1247 | | |  | W3: N = 1247 | | |  | W4: N = 1247 | | | |
| --- | --- | --- | --- | --- | --- | --- | --- | --- | --- | --- | --- | --- | --- | --- | --- | --- |
|  | *M* | *SD* | *NA* |  | *M* | *SD* | *NA* |  | *M* | *SD* | *NA* |  | *M* | *SD* | *NA* |  |
| Age | 7.03 | 0.4 | 9 |  | 7.94 | 0.38 | 56 |  | 8.93 | 0.38 | 67 |  | 11.01 | 0.4 | 429 |  |
| Involvement | 4.19 | 0.42 | 15 |  | 4.1 | 0.42 | 56 |  | 4.08 | 0.41 | 67 |  | 4.02 | 0.44 | 175 |  |
| Positive Parenting | 4.21 | 0.52 | 16 |  | 4.16 | 0.52 | 56 |  | 4.16 | 0.54 | 67 |  | 4.09 | 0.57 | 175 |  |
| Poor Monitoring | 1.31 | 0.33 | 17 |  | 1.35 | 0.35 | 56 |  | 1.37 | 0.36 | 67 |  | 1.48 | 0.42 | 175 |  |
| Inconsistent Discipline | 2.24 | 0.55 | 17 |  | 2.2 | 0.54 | 56 |  | 2.22 | 0.55 | 68 |  | 2.24 | 0.54 | 175 |  |
| Corporal Punishment | 1.4 | 0.4 | 1 |  | 1.38 | 0.46 | 57 |  | 1.35 | 0.46 | 68 |  | 1.25 | 0.43 | 175 |  |

**B)**

|  | W5: N = 1482 | | |  | W6: N = 1482 | | |  | W7: N = 1482 | | |  |
| --- | --- | --- | --- | --- | --- | --- | --- | --- | --- | --- | --- | --- |
|  | *M* | *SD* | *NA* |  | *M* | *SD* | *NA* |  | *M* | *SD* | *NA* |  |
| Age | 13.67 | 0.36 | 117 |  | 15.44 | 0.36 | 36 |  | 17.45 | 0.37 | 176 |  |
| Involvement | 3.08 | 0.59 | 120 |  | 3.01 | 0.62 | 36 |  | 2.96 | 0.63 | 181 |  |
| Positive Parenting | 3.2 | 0.6 | 121 |  | 3.09 | 0.65 | 36 |  | 2.99 | 0.67 | 181 |  |
| Poor Monitoring | 1.89 | 0.51 | 120 |  | 1.9 | 0.51 | 36 |  | 2.03 | 0.54 | 181 |  |
| Inconsistent Discipline | 2.25 | 0.67 | 121 |  | 2.28 | 0.7 | 36 |  | 2.16 | 0.69 | 190 |  |
| Corporal Punishment | 1.19 | 0.41 | 121 |  | 1.16 | 0.38 | 36 |  | 1.13 | 0.36 | 190 |  |

***Note.*** **A)** Behavioral data from assessment waves (W) 1 to 4. Parenting behaviors were measured with the Alabama Parenting Questionnaire (APQ)^2^. Scores represent averages of items rated on a 5-point Likert-type scale (possible score range: 1-5, from low to high). **B)** Behavioral data from assessment waves (W) 5 to 7. Parenting behaviors were measured with an adapted version of the APQ (see Table S6). Scores represent averages of items rated on a 4-point Likert-type scale (possible score range: 1-4, from low to high). *NA* = number of children with missing data in the respective variable.

**Table S2*.*** Study cohorts and available data at each assessment wave (W)

|  |  | **Cohort 1** |  | **Cohort 2** | | | | | | | | | |
| --- | --- | --- | --- | --- | --- | --- | --- | --- | --- | --- | --- | --- | --- |
| Age (years) |  | W1  10 |  | W1  7 | W2  8 | W3  9 | W4  11 |  | W5^*^  14 | W6  15 | W7  17 |  | **MRI subgroup**  22 |
| Parenting reports |  | 41 |  | 1247^a^ | 1247^a^  (1192 available; 55 [4%] imputed) | 1247^a^  (1180 available; 67 [5%] imputed) | 1247^a^  (1075 available; 172 [14%] imputed);  1147^b^;  1058^c^ |  | 1482^b^ | 1482^b^ (1446 available; 36 [2%] imputed) | 1482^b^ (1305 available; 177 [12%] imputed) |  | - |
| sMRI |  | 42 |  |  |  |  |  |  |  |  |  |  | 142 |
| Overlap  Parenting & sMRI |  | 41 |  |  |  |  |  |  |  |  |  |  | 135 (W1-W4)  140 (W5-W7) |
| Exclusion |  | -1 (cognitive delay) |  |  |  |  |  |  |  |  |  |  | -1 (transgender who takes hormones) |
| **Final** |  | **Aim 2.1: 40** (17 females, 23 males) |  | **Aim 1.1:** **1247**^a^ (600 females, 647 males) | **1482**^b^  (715 females, 767 males) |  | **Aim 1.2:** 1058 (513 females, 545 males) |  |  |  |  |  | **Aim 2.2: 134** (full; 51 females, 83 males)  **40** (matched; 17 females, 23 males)  **126** (sample with CP across W1 to W7) |

*Note.* ^*^Rerecruitment efforts led to an increased participation number at W5. CP = corporal punishment; W = wave.

^a^Parental reports of parenting behaviors

^b^Adolescents’ reports of parenting behaviors

^c^ Reports available from both parents and adolescents

**Table S3*.*** Parental education: transformation of values between cohorts

| **Raw cohort 1 score** | **ISCED score cohort 1** | **ISCED label cohort 1** | **Label cohort 2** | **Final parental education value** |
| --- | --- | --- | --- | --- |
|  | 0 | Early childhood education | - | 1 |
|  | 1 | Primary education | Incomplete compulsory school | 1 |
| 1 (Compulsory school completed) | 2 | Lower secondary education | Compulsory school, elementary vocational training | 2 |
| 2 (Matura = A-Levels) | 3 | Matura / Upper secondary education | A-Levels | 6 |
| 6 (Fachhochschule = Higher specialized school)  3 (Bachelor) | 6  6  6 | Berufsschulabschluss = Apprenticeship  Fachhochschule = Higher specialized school  Bachelor | Apprenticeship  Vocational high school, higher specialized school  University / ETH | 4  9  10 |
| 4 (Master) | 7 | Master | University / ETH | 10 |
| 5 (PhD) | 8 | PhD | University / ETH | 10 |
| 7 (Other) |  |  |  |  |

***Note:*** ISCED *=* International Standard Classification of Education^32^; ETH = “Eidgenössische Technische Hochschule” (Federal Institute of Technology).

**Table S4*.*** Parenting behaviors: Reliabilities/ internal consistency – cohort 1

| *Parenting Scale* | Nr of items | α | ω |
| --- | --- | --- | --- |
| Involvement | 10 | 0.48 | 0.61 |
| Positive Parenting | 6 | 0.62 | 0.67 |
| Poor Monitoring | 10 | 0.74 | 0.75 |
| Inconsistent Discipline | 6 | 0.7 | 0.74 |
| Corporal Punishment | 3 | 0.39 | - |
| Composite Adaptive Parenting | 16 | 0.68 | 0.76 |
| Composite Maladaptive Parenting | 19 | 0.76 | - |

***Note.*** N = 40; α = Cronbach’s alpha; ω = McDonald’s omega (- if omega could not be estimated reliably).

**Table S5*.*** Parenting behaviors: Reliabilities/ internal consistency – cohort 2

**A)**

|  |  | W1 | |  | W2 | |  | W3 | |  | W4 | |
| --- | --- | --- | --- | --- | --- | --- | --- | --- | --- | --- | --- | --- |
|  | *Nr* of items | α | ω |  | α | ω |  | α | ω |  | α | ω |
| Involvement | 10 | 0.63 | 0.66 |  | 0.68 | 0.7 |  | 0.66 | 0.69 |  | 0.68 | 0.7 |
| Positive Parenting | 5 | 0.56 | 0.63 |  | 0.61 | 0.68 |  | 0.67 | 0.72 |  | 0.61 | 0.66 |
| Poor Monitoring | 10 | 0.64 | 0.67 |  | 0.68 | 0.7 |  | 0.74 | 0.76 |  | 0.74 | 0.76 |
| Inconsistent Discipline | 6 | 0.52 | 0.55 |  | 0.56 | 0.59 |  | 0.58 | 0.62 |  | 0.54 | 0.6 |
| Corporal Punishment | 3 | 0.53 | 0.57 |  | 0.54 | 0.58 |  | 0.55 | 0.6 |  | 0.62 | 0.64 |
| Comp Adaptive Parenting | 15 | 0.69 | 0.74 |  | 0.75 | 0.78 |  | 0.76 | 0.78 |  | 0.75 | 0.77 |
| Comp Maladaptive Parenting | 19 | 0.64 | 0.67 |  | 0.7 | 0.72 |  | 0.71 | 0.74 |  | 0.73 | 0.76 |

**B)**

|  |  | W5 | |  | W6 | |  | W7 | |
| --- | --- | --- | --- | --- | --- | --- | --- | --- | --- |
|  | *Nr* of items | α | ω |  | α | ω |  | α | ω |
| Involvement | 6 | 0.75 | 0.75 |  | 0.77 | 0.77 |  | 0.77 | 0.78 |
| Positive Parenting | 3 | 0.65 | 0.66 |  | 0.72 | 0.72 |  | 0.72 | 0.73 |
| Poor Monitoring | 7 | 0.68 | 0.69 |  | 0.67 | 0.69 |  | 0.66 | 0.69 |
| Inconsistent Discipline | 3 | 0.47 | 0.48 |  | 0.52 | 0.53 |  | 0.48 | 0.52 |
| Corporal Punishment | 3 | 0.71 | 0.72 |  | 0.73 | 0.74 |  | 0.74 | 0.76 |
| Comp Adaptive Parenting | 9 | 0.8 | 0.81 |  | 0.83 | 0.83 |  | 0.83 | 0.84 |
| Comp Maladaptive Parenting | 13 | 0.64 | 0.66 |  | 0.63 | 0.65 |  | 0.57 | 0.62 |

**C)**

|  |  | W1 | |  | W2 | |  | W3 | |  | W4 | |
| --- | --- | --- | --- | --- | --- | --- | --- | --- | --- | --- | --- | --- |
|  | *Nr* of items | α | ω |  | α | ω |  | α | ω |  | α | ω |
| Involvement | 10 | 0.59 | 0.6 |  | 0.68 | 0.71 |  | 0.65 | 0.68 |  | 0.69 | 0.68 |
| Positive Parenting | 5 | 0.65 | 0.71 |  | 0.57 | 0.66 |  | 0.70 | 0.75 |  | 0.74 | 0.79 |
| Poor Monitoring | 10 | 0.69 | 0.72 |  | 0.7 | 0.73 |  | 0.77 | 0.79 |  | 0.8 | 0.81 |
| Inconsistent Discipline | 6 | 0.4 | 0.47 |  | 0.4 | 0.45 |  | 0.55 | 0.64 |  | 0.5 | 0.55 |
| Corporal Punishment | 3 | 0.51 | 0.54 |  | 0.58 | 0.61 |  | 0.55 | 0.64 |  | 0.64 | 0.7 |
| Comp Adaptive Parenting | 15 | 0.72 | 0.76 |  | 0.73 | 0.77 |  | 0.75 | 0.77 |  | 0.78 | 0.78 |
| Comp Maladaptive Parenting | 19 | 0.54 | 0.66 |  | 0.6 | 0.66 |  | 0.69 | 0.73 |  | 0.74 | 0.76 |

**D)**

|  |  | W5 | |  |
| --- | --- | --- | --- | --- |
|  | *Nr* of items | α | ω |  |
| Involvement | 6 | 0.66 | 0.67 |  |
| Positive Parenting | 3 | 0.62 | 0.63 |  |
| Poor Monitoring | 7 | 0.61 | 0.63 |  |
| Inconsistent Discipline | 3 | 0.43 | 0.46 |  |
| Corporal Punishment | 3 | 0.65 | 0.7 |  |
| Comp Adaptive Parenting | 9 | 0.76 | 0.78 |  |
| Comp Maladaptive Parenting | 13 | 0.58 | 0.6 |  |

***Note.*** **A)** Internal consistencies of parent-reported parenting behaviors. **B)** Internal consistencies of child/adolescent-reported parenting behaviors. Neuroimaging subgroup: **C)** Internal Consistencies for parent-reported parenting behaviors of the neuroimaging group of cohort 2. **D)** Internal consistencies of child/adolescent-reported parenting behaviors: W5 data was used for some children in the matched group of cohort 2. Comp = Composite Score; α = Cronbach’s alpha; ω = McDonald’s omega

**Table S6*.*** Parenting questionnaire’s instructions and items for adolescents’ reports (age 11 to 17, cohort 1)

| **I/Q** | **Item (English)**  *Item (German)* | **APQ Scale** | **11y** | **14y** | **15y** | **17y** |
| --- | --- | --- | --- | --- | --- | --- |
| I | **You and your parents**  **Please mark with crosses which of the things *below occur never, rarely, sometimes* or *often* in your home.**  **^b)c)^“*Parents“ are the adults that care for you at home.***  **^c)^ *If you no longer live with your parents and therefore can’t answer certain questions, please just leave those questions blank.***  *Du und deine Eltern*  *Kreuze bitte an, ob das, was in den Sätzen steht, bei dir zu Hause  nie, selten, manchmal oder häufig vorkommt.*  *^b c)^„Eltern“ sind die Erwachsenen, die zu Hause für dich sorgen.*  *^c)^ Wenn du nicht mehr bei deinen Eltern wohnst und deshalb gewisse Fragen nicht beantworten kannst, lässt du diese bitte einfach leer.* |  | X | X^b^ | X^b^ | X^c^ |
| Q | **^a)^Your parents talk to you about your friends or about the other kids in your class.**  **^b)^Your parents talk to you about your friends or about the adolescents in your class.**  *^a)^Deine Eltern reden mit dir über deine Freunde oder über die Kinder in deiner Klasse.*  *^b)^Deine Eltern reden mit dir über deine Freunde oder über die Jugendlichen in deiner Klasse.* | Involvement | x^a^ | x^b^ | x^b^ | x^b^ |
| Q | **You leave your house without telling your parents where you are going.**  *^a)^Du gehst einfach von zu Hause heraus, ohne deinen Eltern zu sagen wohin.*  *^b)^Du gehst einfach von zu Hause weg, ohne deinen Eltern zu sagen wohin.* | Poor Monitoring | x^a^ | x^b^ | x^b^ | x^b^ |
| Q | **Your parents let you know when you have done something well.**  *Wenn du etwas gut gemacht hast, sagen dir das deine Eltern.* | Positive Parenting | x | x | x | x |
| Q | **You play games or do other fun things with your parents.**  *Deine Eltern spielen oder unternehmen etwas zusammen mit dir.* | Involvement | x | x | x | x |
| Q | **You have to tell your parents who you meet in your free time.**  *Du musst deinen Eltern sagen, mit wem du dich in deiner Freizeit triffst.* | Poor Monitoring - reversed |  | x | x | x |
| Q | **You come home in the evening later than agreed.**  *Du kommst am Abend später als abgemacht nach Hause.* | Poor Monitoring | x | x | x | x |
| Q | **Your parents reward you for doing something well.**  *Wenn du etwas gut gemacht hast, belohnen dich deine Eltern dafür.* | Positive Parenting | x | x | x | x |
| Q | **Your parents help you when you struggle with your homework.**  *Deine Eltern helfen dir, wenn du mit den Schulaufgaben Mühe hast.* | Involvement | x | x | x | x |
| Q | **When you come home in the afternoon, there are no adults at home.**  *Wenn du am Nachmittag nach Hause kommst, sind keine Erwachsenen zu Hause.* | Poor Monitoring | x | x | x | x |
| Q | **Your mother or father hugs you to comfort you when you are sad.**  *Wenn du traurig bist, nimmt dich deine Mutter oder dein Vater zum Trost in die Arme.* | Involvement | x | x | x | x |
| Q | **Your parents ask you what you got up to in your free time.**  *Deine Eltern sprechen dich darauf an, was du in deiner Freizeit gemacht hast.* | Poor Monitoring - reversed |  | x | x | x |
| Q | **Your parents show interest in what you do.**  *Deine Eltern interessieren sich für das, was du machst.* | Involvement | x | x | x | x |
| Q | **When you go out in your free time, your parents ask you where you are going.**  *Wenn du in deiner Freizeit ausgehst, fragen dich deine Eltern, wohin du gehst.* | Poor Monitoring - reversed | x | x | x | x |
| Q | **You hide from your parents what you do in the evenings and on weekends.**  *Du verheimlichst deinen Eltern, was du abends und am Wochenende machst.* | Poor Monitoring |  | x | x | x |
| Q | **Your parents compliment you if you were particularly good at school, in a sport, or at a hobby.**  *Deine Eltern loben dich, wenn du in der Schule, im Sport oder bei deinem Hobby besonders gut warst.* | Positive Parenting |  | x | x | x |
| Q | **When you have problems, you can go to your parents.**  *Wenn du Probleme hast, kannst du damit zu deinen Eltern gehen.* | Involvement | x | x | x | x |
| Q | **When you go out in your free time, your parents tell you what time to come home.**  *Wenn du in deiner Freizeit ausgehst, sagen dir deine Eltern, um welche Zeit du heimkommen musst.* | Poor Monitoring - reversed | x | x | x | x |
| I | **^a)^When you misbehave or are disobedient, what do your parents do? Do your parents do the things below either *never*, *rarely*, *sometimes* or *often*?**  *^a)^Wenn du etwas anstellst oder ungehorsam bist, was machen deine Eltern dann? Machen deine Eltern das, was in den Sätzen steht, nie, selten, manchmal oder häufig mit dir?*  **^b)^When you misbehave or are disobedient, what do your parents do? Do your parents do the things below either *never*, *rarely*, *sometimes* or *often*?**  ***If you no longer live with your parents you can simply skip the following questions and leave them blank.***  *^b)^Wenn du etwas anstellst oder ungehorsam bist, was machen deine Eltern dann?*  *Machen deine Eltern das, was unten steht nie, selten, manchmal oder häufig mit dir?*  *Wenn du nicht mehr bei deinen Eltern wohnst, kannst du die folgenden Fragen einfach überspringen und leer lassen.* |  | x^a^ | x^a^ | x^a^ | x^b^ |
| Q | **Your parents yell or scream at you.**  *Deine Eltern schreien dich an.* | Other | x | x | x | x |
| Q | **You talk your parents out of punishing you when you have done something wrong.**  *Deine Eltern lassen sich von dir überreden und bestrafen dich nicht.* | Inconsistent Discipline |  | x | x | x |
| Q | **Your parents calmly explain to you why your behaviour is wrong.**  *Deine Eltern erklären dir ruhig, warum man das nicht tun darf.* | Other | x | x | x | x |
| Q | **Your parents give you extra chores as a punishment. (*e.g. cleaning the dishes, or tidying*)**  *Deine Eltern geben dir zur Strafe eine Arbeit.  z.B. abwaschen oder aufräumen* | Other | x | x | x | x |
| Q | **Your parents threaten to punish you but subsequently do nothing.**  *Deine Eltern drohen dir eine Strafe an, tun dann aber doch nichts.* | Inconsistent Discipline | x | x | x | x |
| Q | **Your parents slap you.**  *Deine Eltern geben dir eine Ohrfeige.* | Corporal Punishment | x | x | x | x |
| Q | **Your parents take away privileges or money as a punishment.**  **(*e.g. forbid you from watching TV, or send you to bed early*)**  *Deine Eltern verbieten dir etwas, das du sonst tun dürftest.  z.B. kein Fernsehen schauen oder früher ins Bett müssen* | Other | x | x | x | x |
| Q | **Your parents reduce the punishment after the fact. *(e.g. you are allowed to watch TV or go out earlier than originally said.*)**  *Deine Eltern verringern im Nachhinein die Strafe. (z.B. darfst du früher als abgemacht wieder fernsehen oder in den Ausgang gehen.)* | Inconsistent Discipline |  | x | x | x |
| Q | **Your parents spank you with their hand.^1^**  *Deine Eltern geben dir eins auf den Hintern.* | Corporal Punishment | x | x | x |  |
| Q | **Your parents punish you more severely than usual when they are in a bad mood.**  *Wenn sie schlecht gelaunt sind, bestrafen dich deine Eltern strenger.* | Inconsistent Discipline | x | x | x | x |
| Q | **Your parents hit you with a belt, staff, or other object.**  *Deine Eltern schlagen dich mit einem Stock, Gürtel oder sonstigem Gegenstand.* | Corporal Punishment |  | x | x | x |
| Q | **Your parents pull your hair or ears.**  *Deine Eltern ziehen dich an den Ohren oder an den Haaren.* | Corporal Punishment | x | x | x | x |
| Q | **Your parents send you to your room.**  *Deine Eltern schicken dich in dein Zimmer.* | Other | x | x | x | x |
| Q | **Your parents do not punish you and leave you alone.**  *Deine Eltern machen nichts und lassen dich in Ruhe.* | Inconsistent Discipline |  | x | x | x |

***Note.*** Items at wave 4 to wave 7 (age 11, 14, 15, 17). Items are adapted from the Alabama Parenting Questionnaire (APQ). Rating scale ranges from 1 (“never”) to 4 (“often”). To enhance compliance corporal punishment and inconsistent discipline items were asked in a separate second part, which also included some distractor items (“Other”). I = instruction; Q = question. Table is adapted with permission of authors from^24^.

^1^ This item was not included in the longitudinal analyses (behavioral variations of parenting over time) as it was not asked at age 17.

**Table S7*.*** Descriptives of composite parenting scores across all neuroimaging (sub)groups

|  |  | Cohort 1  (*N* = 40) | |  | Cohort 2 (N = 134):  Neuroimaging Subgroup  Wave 1 Wave 2 Wave 3 Wave 4 Wave 5 | | | | | | | | | |  | Cohort 2: Matched Neuroimaging Subgroup  (N = 40) | |
| --- | --- | --- | --- | --- | --- | --- | --- | --- | --- | --- | --- | --- | --- | --- | --- | --- | --- |
| *Parenting Behaviors* |  | M | SD |  | M | SD | M | SD | M | SD | M | SD | M | SD |  | M | SD |
| Adaptive Parenting |  | 3.9 | 0.3 |  | 4.2 | 0.4 | 4.1 | 0.4 | 4.1 | 0.4 | 4 | 0.4 | 3.1 | 0.5 |  | 3.9 | 0.3 |
| Maladaptive Parenting |  | 1.7 | 0.3 |  | 1.7 | 0.3 | 1.7 | 0.3 | 1.7 | 0.3 | 1.7 | 0.3 | 1.7 | 0.3 |  | 1.8 | 0.3 |

**
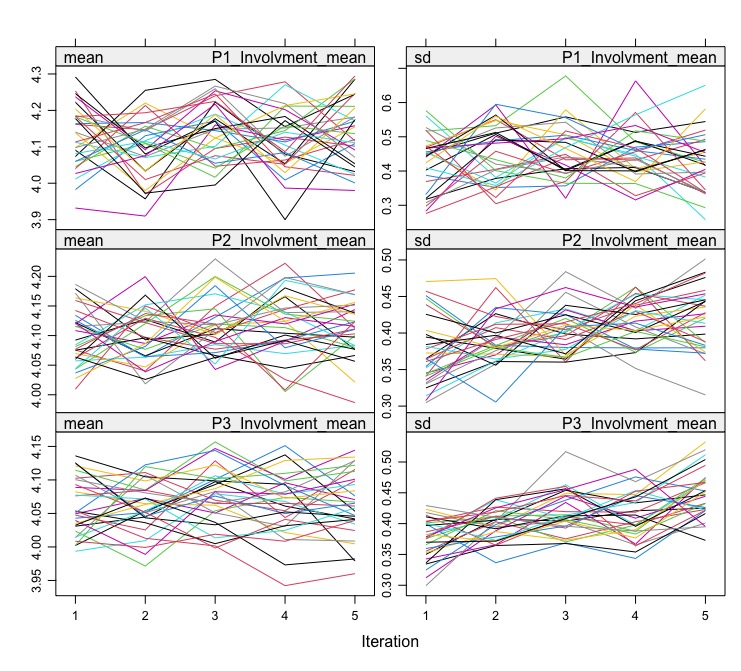

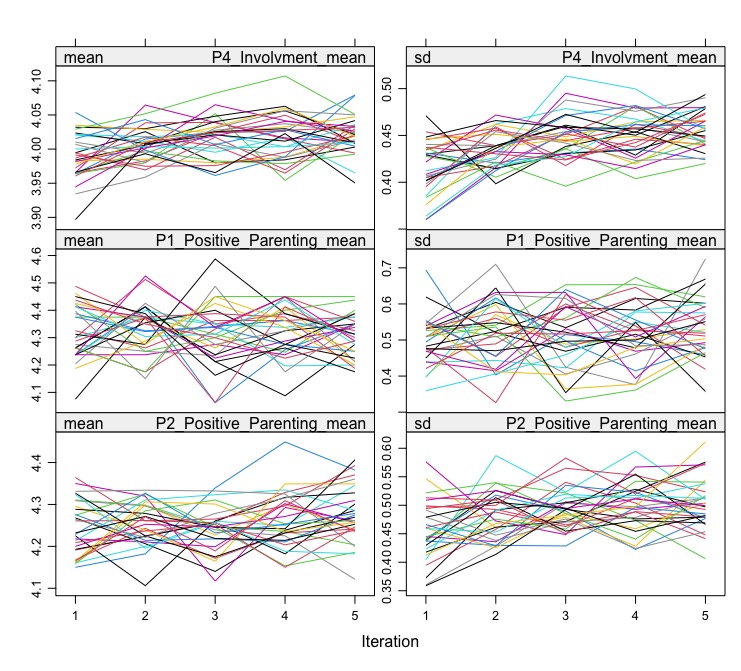
**

### Figure S1. Representative MCMC trace plots for imputation diagnostics (Involvement subscale, W1-W4). Trace plots displaying the mean (left) and standard deviation (right) for the Involvement subscale across the 30 imputed datasets. The five MCMC iterations demonstrate that the chains achieved stationarity and excellent mixing, confirming successful convergence of the imputation algorithm. P1-P4 denote the assessment time points for parent-rated parenting behaviors (child ages 7-11 years). MCMC = Markov Chain Monte Carlo; W = assessment wave.

**A)**

**
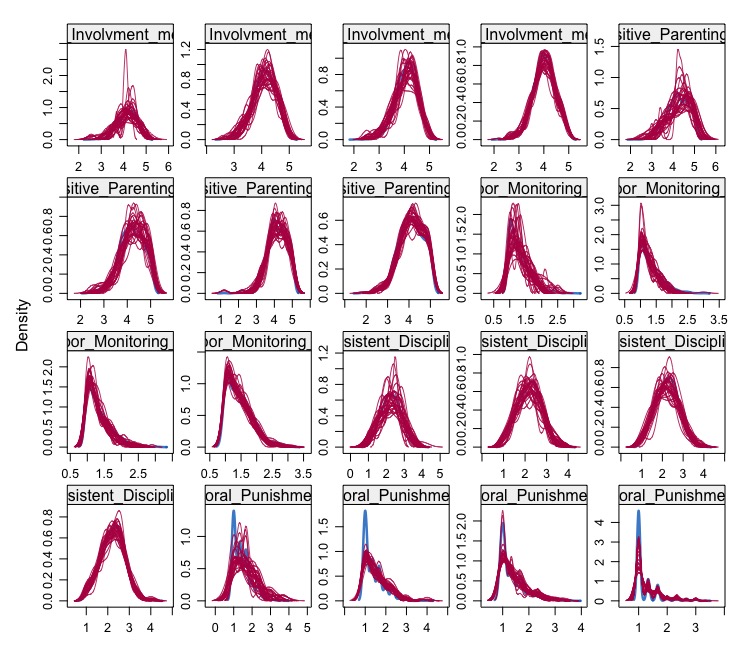
**

**B)**

**
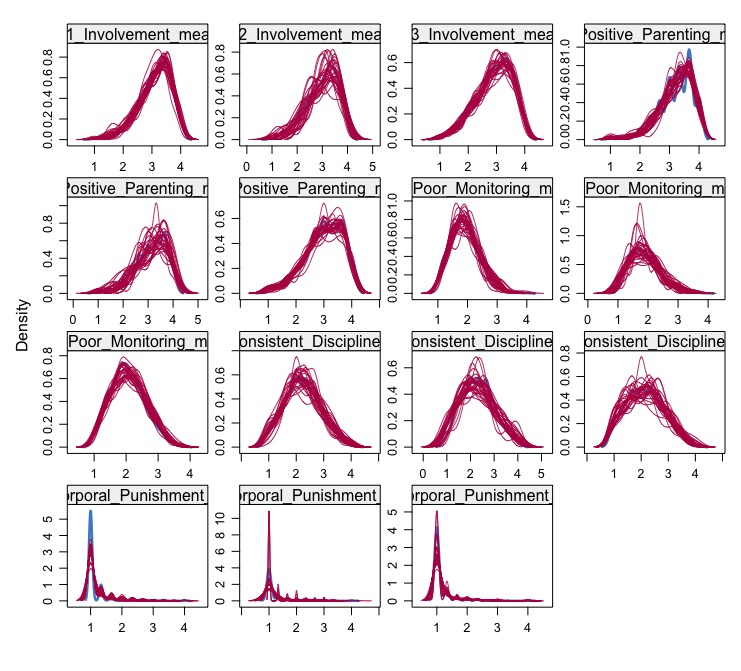
**

**Figure S2.** Density overlays of observed and imputed data. **A)** Assessment waves W1-W4 (parent-reported parenting behaviors. B) Assessment waves W5-W7 (child/adolescent-reported parenting behaviors).

**Table S8.** Stability and reliability of parenting behaviors across time points (7-11 years) in the neuroimaging group of cohort 2

| **APQ Scale** | **ICC3** | **ICC3k** | ***r*_s​_ range** |
| --- | --- | --- | --- |
| Involvement | .57 | .84 | .50-.69 |
| Positive Parenting | .64 | .88 | .61-.68 |
| Poor Monitoring | .65 | .88 | .50-.72 |
| Inconsistent Discipline | .50 | .80 | .40-.52 |
| Corporal Punishment | .62 | .87 | .49-.74 |

**Note. N = 127-128 with complete items across timepoints. Values refer to parenting behaviors across 4 time points (parent-reported parenting behaviors from child age 7, 8, 9 and 11 years); ICC = Intraclass correlation coefficient; ICC3 = Single-Measure ICC; ICC3k = Average-Measure ICC; *r*_s​_ = Spearman rank-order correlation.**

**Table S9.** Significant regressions of cohort 1 with parental education added as a proxy for SES

| *Regressors* | $\beta$ | | (95% CI) | t | *p* | R*^2^*_adj_ |  | $\beta$ | | (95% CI) | t | *p* | R^2^_adj_ |
| --- | --- | --- | --- | --- | --- | --- | --- | --- | --- | --- | --- | --- | --- |
|  | *Right Amygdala* | | | | | |  | *Left dlPFC*  *CT* | | | | | |
| (Intercept) | 0 | (-0.20, 0.20) | | 0 | 1 | .45^a^  .61^b^ |  | 0 | (-0.26, 0.26) | | 0 | 1 | .20^a^  .37^b^ |
| Sex | 0.14 | (-0.12, 0.41) | | 1.12 | .271 |  |  | 0.21 | (-0.07, 0.49) | | 1.53 | .136 |  |
| Age | -0.02 | (-0.26, 0.21) | | -0.21 | .837 |  |  | -0.24 | (-0.54, 0.06) | | -1.65 | .11 |  |
| TIV | 0.73 | (0.48, 0.99) | | 5.87 | <.001 |  |  | - | - | | - | - |  |
| Parental education | -0.08 | (-0.34, 0.18) | | -0.63 | .534 |  |  | -0.18 | (-0.5, 0.14) | | -1.14 | .262 |  |
| Involvement | 0.07 | (-0.15, 0.28) | | 0.63 | .536 |  |  | 0.21 | (-0.06, 0.48) | | 1.56 | .129 |  |
| Positive Parenting | 0.51 | (0.26, 0.77) | | 4.11 | <.001* |  |  | 0 | (-0.32, 0.33) | | 0.02 | .983 |  |
| Poor Monitoring | -0.04 | (-0.28, 0.2) | | -0.35 | .725 |  |  | -0.17 | (-0.47, 0.13) | | -1.17 | .252 |  |
| Inconsistent Discipline | 0.03 | (-0.21, 0.26) | | 0.23 | .823 |  |  | 0.12 | (-0.18, 0.42) | | 0.82 | .417 |  |
| Corporal Punishment | 0.13 | (-0.13, 0.39 | | 0.99 | .331 |  |  | -0.51 | (-0.84, -0.18) | | -3.17 | .003* |  |

***Note:*** Only the model predicting grey matter volume (GMV) included total intracranial volume (TIV) as covariate. *Significant on adjusted significance level of *p* < 0.00625 (Bonferroni correction for multiple comparison); CT = cortical thickness; $\beta$ = standardized beta; SE B = standard error for the unstandardized beta; R^2^_adj_ = adjusted R^2^ of ^a^models with control regressors only vs. ^b^full models; dlPFC = dorsolateral prefrontal cortex; TIV = total intracranial volume.

**Table S10.** Variance inflation factors (VIF) of cohort 1 regressors

| *Regressors* | *GMV outcomes* | *GMV outcomes*  *with Parental Education included* | *CT*  *outcomes* | *CT outcomes*  *with Parental Education included* |
| --- | --- | --- | --- | --- |
| Sex | 1.63 | 1.65 | 1.15 | 1.16 |
| Age | 1.34 | 1.34 | 1.32 | 1.33 |
| TIV | 1.47 | 1.55 | **-** | **-** |
| Involvement | 1.13 | 1.14 | 1.11 | 1.12 |
| Positive Parenting | 1.42 | 1.55 | 1.41 | 1.55 |
| Poor Monitoring | 1.32 | 1.34 | 1.32 | 1.34 |
| Inconsistent Discipline | 1.31 | 1.33 | 1.29 | 1.32 |
| Corporal Punishment | 1.40 | 1.61 | 1.40 | 1.6 |
| Parental Education | - | 1.59 | - | 1.5 |

***Note:*** Only the models predicting GMV included total intracranial volume (TIV) as covariate. CT = cortical thickness; GMV = grey matter volume.

**
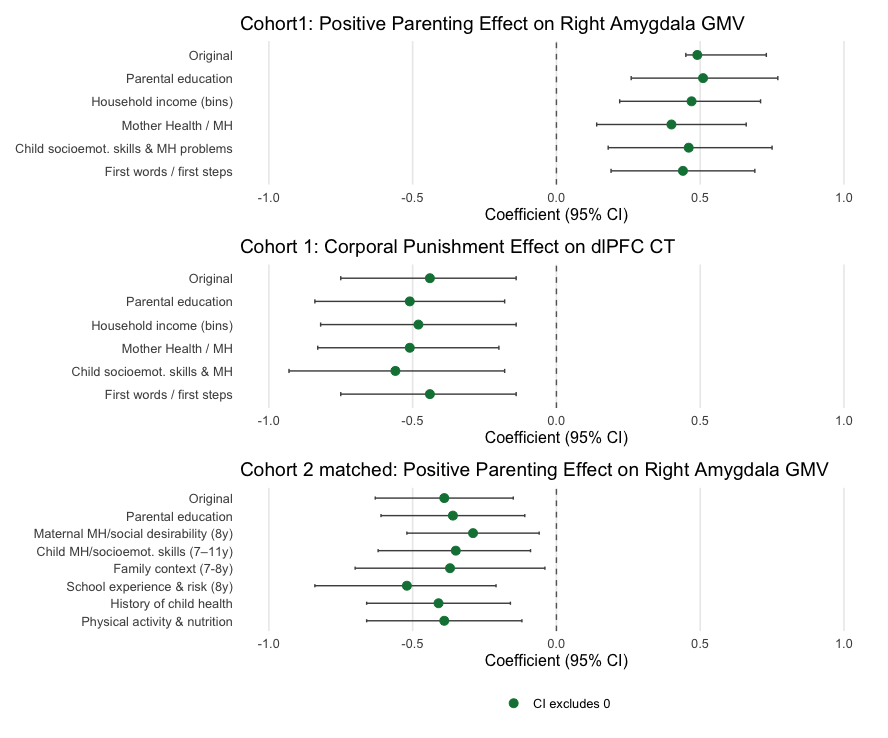
**

**Figure S3.** Multiple sensitivity analyses for all significant parenting behavior-brain associations. MH = mental health. GMV = grey matter volume; dlPFC CT = dorso lateral prefrontal cortical thickness.

**Table S11.** Multiple sensitivity analyses for all significant parenting behavior-brain associations

**A) Cohort 1: Positive Parenting and right amygdala volume**

|  | $\beta$ | CI low | CI high | VIF |
| --- | --- | --- | --- | --- |
| Original | 0.49 | 0.45 | 0.73 | 1.42 |
| Parental education | 0.51 | 0.26 | 0.77 | 1.55 |
| Household income (bins) | 0.47 | 0.22 | 0.71 | 1.42 |
| Mother Health / MH | 0.40 | 0.14 | 0.66 | 1.91 |
| Child Socioemot.  Skills & mental health problems | .46 | 0.18 | 0.75 | 1.75 |
| First Words / first steps | 0.44 | 0.19 | 0.69 | 1.52 |

**B) Cohort 1: Corporal Punishment and left dlPFC thickness**

|  | $\beta$ | CI low | CI high | VIF |
| --- | --- | --- | --- | --- |
| Original | -0.44 | -.75 | 0.14 | 1.40 |
| Parental education | -0.51 | -0.84 | -0.18 | 1.60 |
| Household income (bins) | -0.48 | -0.82 | -0.14 | 1.62 |
| Mother Health / MH | -0.51 | -0.83 | -0.20 | 1.71 |
| Child Socioemot.  Skills & Mental health | -0.56 | -0.93 | -0.18 | 1.89 |
| First Words / first steps | -0.44 | -0.75 | -0.14 | 1.45 |

**C) Cohort 2 matched group: Positive Parenting and right amygdala volume**

|  | $\beta$ | CI low | CI high | VIF |
| --- | --- | --- | --- | --- |
| Original | -0.39 | -0.63 | -0.15 | 1.62 |
| Parental education | -0.36 | -0.61 | -0.11 | 1.73 |
| Maternal depression, self-control, social desirability (available at 8y) | -0.29 | -0.52 | -0.06 | 1.81 |
| Child mental difficulties/Socioemot.  Skills (internalizing, externalizing, prosocial behavior across 7-11y) | -0.35 | -0.62 | -0-09 | 1.85 |
| Parent Problem Checklist (8y) – Disagreement between parents; nr of siblings at 7y | -0.37 | -0.70 | -0.04 | 1.53^a^ |
| School experience: child likes school, teacher reported school problems, violent peers at 8y and cumulative school risk | -0.52 | -0.84 | -0.21 | 1.86 |
| Severe complications during pregnancy or birth, alcohol consumption during pregnancy, How many times injured with doctor visit from birth to study start | -0.41 | -0.66 | -0.16 | 1.70^a^ |
| Physical activity & nutrition per week (in organized sport activities / eats fruits/veggies between meals) | -0.39 | -0.66 | -0.12 | 1.91 |

Note. ^a^ scaled VIF value as factors were included in the models. CI = 95% confidence interval; VIF = variable infaltion factor.


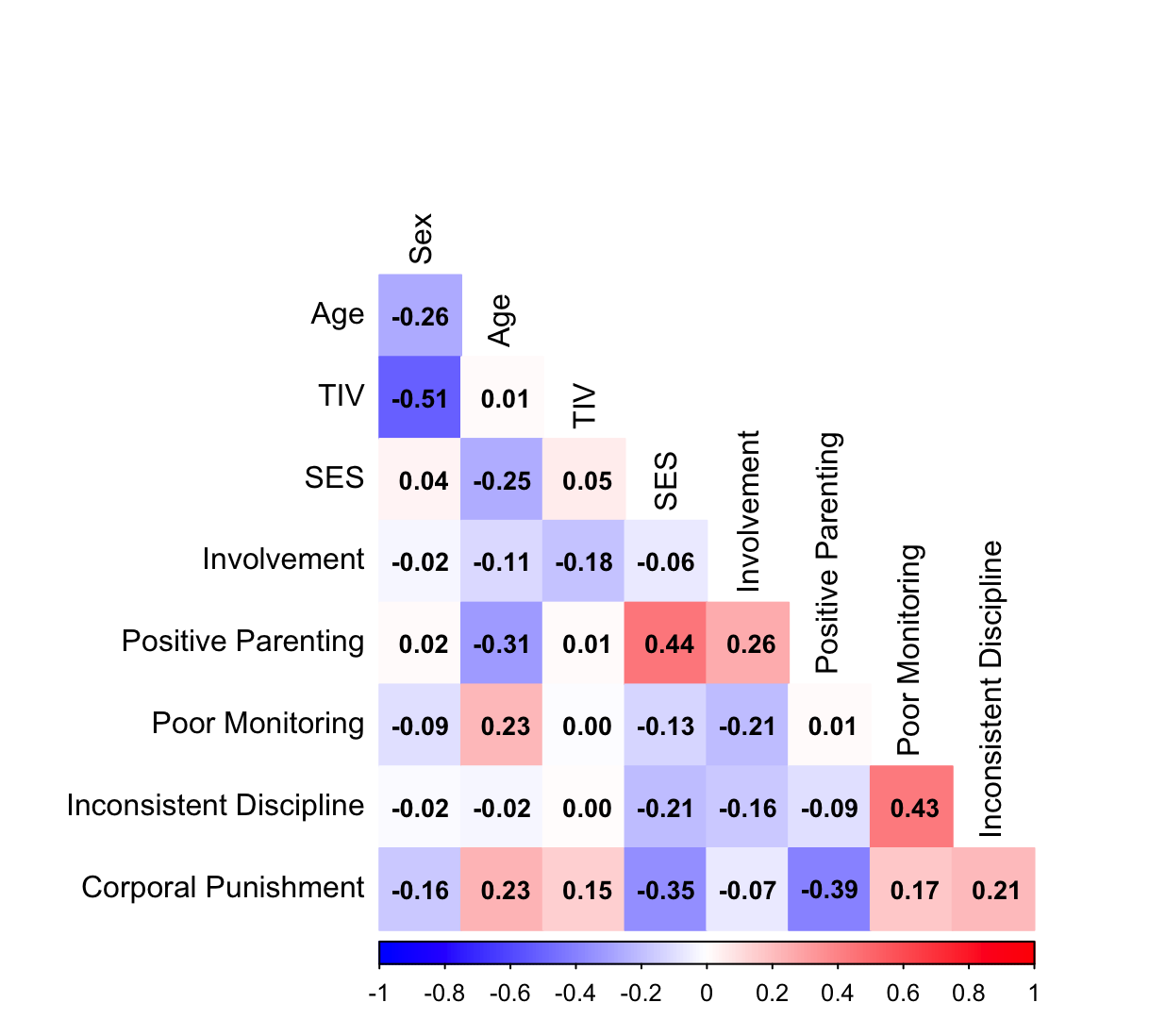


**Figure S4.** Spearman correlation coefficients between all regressors of cohort 1 models. Regressors of interest: parenting behaviors. Control regressors: sex, age, TIV and parental education (SES). TIV = total intracranial volume; SES = Parental education used as a proxy for socioeconomic status.

**Table S12.** Regressions of parenting behaviors on brain GMV and dlPFC CT in late adolescents/young adults.

| *Regressors* | $\beta$ (95% CI) | | t | *p* | R*^2^*_adj_ |  | $\beta$ | (95% CI) | t | *p* | R^2^_adj_ |
| --- | --- | --- | --- | --- | --- | --- | --- | --- | --- | --- | --- |
|  | *Right dlPFC*  *GMV* | | | | |  | *Left dlPFC*  *GMV* | | | | |
| (Intercept) | 0 | (-0.14, 0.14) | 0 | 1 |  |  | 0 | (-0.15, 0.15) | 0 | 1 |  |
| Sex | -0.29 | (-0.47, -0.11) | -3.15 | .002 |  |  | -0.27 | (-0.45, -0.08) | -2.84 | .005 |  |
| TIV | 0.36 | (0.18, 0.54) | 3.94 | <.001 | .31^a^ |  | 0.33 | (0.15, 0.52) | 3.6 | <.001 | .27 ^a^ |
| Involvement | -0.02 | (-0.19, 0.15) | -0.19 | .846 |  |  | 0.01 | (-0.17, 0.18) | 0.07 | .946 |  |
| Positive Parenting | -0.01 | (-0.19, 0.17) | -0.14 | .886 |  |  | -0.02 | (-0.2, 0.16) | -0.23 | .822 |  |
| Poor Monitoring | -0.08 | (-0.24, 0.07) | -1.05 | .296 |  |  | -0.06 | (-0.22, 0.10) | -0.74 | .462 |  |
| Inconsistent Discipline | 0.05 | (-0.10, 0.21) | 0.67 | .502 |  |  | 0.09 | (-0.07, 0.25) | 1.09 | .277 |  |
| Corporal Punishment | -0.09 | (-0.26, 0.08) | -1.05 | .296 | .30^b^ |  | -0.16 | (-0.33, 0.01) | -1.82 | .071 | .27 ^b^ |
|  | *Right dlPFC*  *CT* | | | | |  | *Left dlPFC*  *CT* | | | | |
| (Intercept) | 0 | (-0.17, 0.17) | 0 | 1 |  |  | 0 | (-0.17, 0.17) | 0 | 1 |  |
| Sex | 0 | (-0.17, 0.18) | 0.03 | .975 | -.01 ^a^ |  | -0.05 | (-0.23, 0.13) | -0.59 | .557 | -.00 ^a^ |
| Involvement | -0.04 | (-0.24, 0.17) | -0.37 | .714 |  |  | -0.07 | (-0.28, 0.13) | -0.69 | .492 |  |
| Positive Parenting | 0.09 | (-0.12, 0.31) | 0.88 | .382 |  |  | 0.11 | (-0.10, 0.33) | 1.03 | .303 |  |
| Poor Monitoring | 0.07 | (-0.12, 0.25) | 0.69 | .49 |  |  | 0.02 | (-0.17, 0.21) | 0.24 | .812 |  |
| Inconsistent Discipline | 0.19 | (0, 0.38) | 2 | .047 |  |  | 0.09 | (-0.10, 0.28) | 0.94 | .348 |  |
| Corporal Punishment | -0.11 | (-0.31, 0.1) | -1.04 | .299 | <.01 ^b^ |  | -0.05 | (-0.25, 0.16) | -0.44 | .663 | -.02 ^b^ |
|  | *Right Hippocampus* | | | | |  | *Left Hippocampus* | | | | |
| (Intercept) | 0 | (-0.14, 0.14) | 0 | 1 |  |  | 0 | (-0.14, 0.14) | 0 | 1 |  |
| Sex | -0.25 | (-0.42, -0.08) | -2.89 | .005 |  |  | -0.3 | (-0.48, -0.12) | -3.25 | .002 |  |
| TIV | 0.43 | (0.26, 0.6) | 4.99 | <.001 | .37 ^a^ |  | 0.33 | (0.15, 0.51) | 3.6 | <.001 | .30 ^a^ |
| Involvement | -0.01 | (-0.17, 0.15) | -0.1 | .921 |  |  | -0.07 | (-0.24, 0.11) | -0.75 | .452 |  |
| Positive Parenting | 0.06 | (-0.11, 0.23) | 0.7 | .486 |  |  | 0.09 | (-0.09, 0.27) | 0.97 | .333 |  |
| Poor Monitoring | 0.02 | (-0.13, 0.17) | 0.27 | .790 |  |  | -0.04 | (-0.20, 0.11) | -0.56 | .576 |  |
| Inconsistent Discipline | 0.16 | (0.01, 0.31) | 2.08 | .040 |  |  | 0.1 | (-0.06, 0.26) | 1.27 | .207 |  |
| Corporal Punishment | -0.11 | (-0.28, 0.05) | -1.4 | .163 | .37 ^b^ |  | -0.16 | (-0.33, 0.01) | -1.91 | .058 | .30 ^b^ |
|  | *Right Amygdala* | | | | |  | *Left Amygdala* | | | | |
| (Intercept) | 0 | (-0.14, 0.14) | 0 | 1 |  |  | 0 | (-0.16, 0.16) | 0 | 1 |  |
| Sex | -0.38 | (-0.56, -0.2) | -4.13 | <.001 |  |  | -0.3 | (-0.5, -0.11) | -3.03 | .003 |  |
| TIV | 0.24 | (0.06, 0.42) | 2.62 | .01 | .31 ^a^ |  | 0.18 | (-0.01, 0.38) | 1.87 | .064 | .17 ^a^ |
| Involvement | -0.11 | (-0.29, 0.06) | -1.31 | .192 |  |  | -0.13 | (-0.31, 0.06) | -1.33 | .187 |  |
| Positive Parenting | 0.13 | (-0.05, 0.31) | 1.42 | .157 |  |  | 0.07 | (-0.12, 0.27) | 0.75 | .452 |  |
| Poor Monitoring | 0 | (-0.15, 0.16) | 0.04 | .970 |  |  | -0.09 | (-0.26, 0.08) | -1.02 | .308 |  |
| Inconsistent Discipline | 0.06 | (-0.1, 0.21) | 0.71 | .479 |  |  | 0.13 | (-0.04, 0.30) | 1.51 | .134 |  |
| Corporal Punishment | -0.01 | (-0.18, 0.16) | -0.1 | .923 | .30 ^b^ |  | -0.04 | (-0.23, 0.14) | -0.44 | .658 | .17 ^b^ |

***Note.*** Variables from the Alabama Parenting Questionnaire averaged across W1 to W4 (age 7 to 11 years). GMV = grey matter volume; dlPFC = dorsolateral prefrontal cortex; CT = cortical thickness; *β* = standardized beta; SE B = standard error for the unstandardized beta; R^2^_adj_ = adjusted R^2^ of ^a^models with control regressors only vs. ^b^full models; TIV = total intracranial volume; W = assessment wave. Adjusted significance level of *p* < .008 (Bonferroni correction for multiple comparison)

**Table S13.** VIF of cohort 2 regressors

|  | Cohort 2 (N = 134):  Neuroimaging Subgroup | |  | Cohort 2: Matched Neuroimaging Subgroup  (N = 40) | | |
| --- | --- | --- | --- | --- | --- | --- |
| *Regressors* | *GMV outcomes* | *CT outcomes* |  | *GMV outcomes* | *GMV outcomes*  *with Parental Education included* | *CT outcomes* |
| Sex | 1.61 | 1.05 |  | 1.51 | 1.51 | 1.12 |
| Age | - | **-** |  | 1.40 | 1.42 | 1.38 |
| TIV | 1.57 | **-** |  | 1.68 | 1.68 | **-** |
| Involvement | 1.43 | 1.42 |  | 1.88 | 1.89 | 1.42 |
| Positive Parenting | 1.55 | 1.54 |  | 1.62 | 1.73 | 1.54 |
| Poor Monitoring | 1.18 | 1.18 |  | 1.36 | 1.48 | 1.18 |
| Inconsistent Discipline | 1.2 | 1.2 |  | 1.10 | 1.14 | 1.20 |
| Corporal Punishment | 1.41 | 1.39 |  | 1.09 | 1.19 | 1.39 |
| Parental Education | - | - |  | - | 1.44 | - |

***Note:*** Only the models predicting GMV included total intracranial volume (TIV) as covariate. Age was included as control regressor in the matched group only. Cohort 2 full neuroimaging subgroup (N = 134): parenting behavior scores are based on the average across 4 assessment waves (age 7, 8, 9 and 11 years). For significant associations the regression models were rerun with Parental Education. VIF = Variance Inflation Factor; CT = cortical thickness; GMV = grey matter volume.

**A) B)**


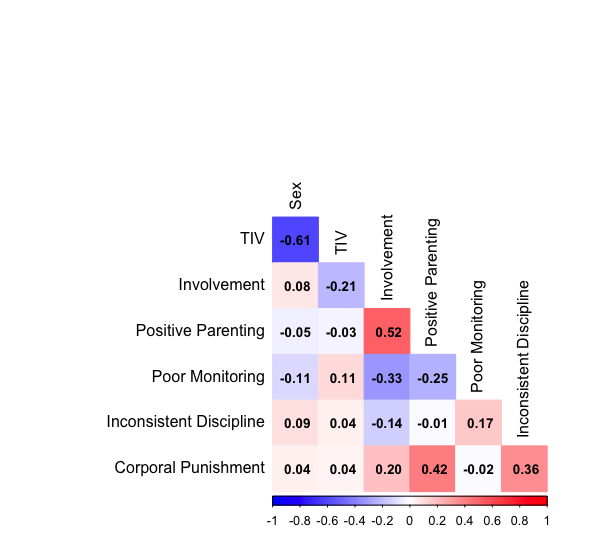

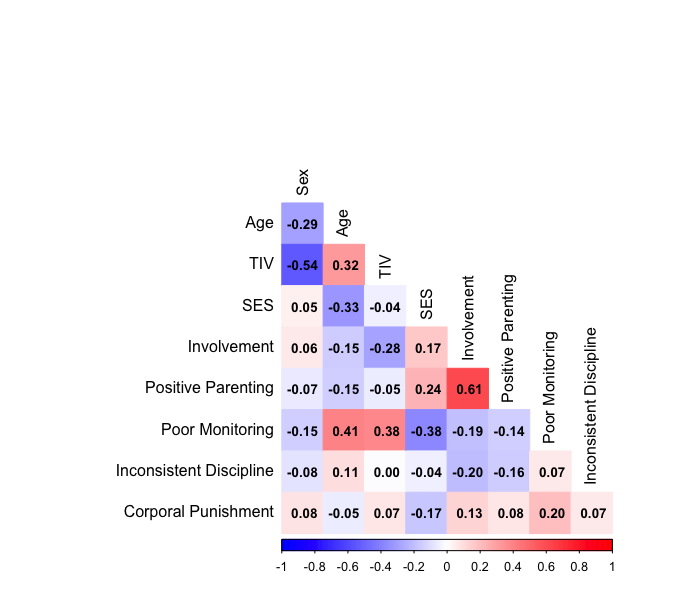
**Figure S5.** Spearman correlation coefficients between all regressors of cohort 2 models. A) Full neuroimaging subgroup cohort 2 (N = 134): Regressors of interest: parenting behaviors averaged across waves 1 to 4 (ages 7, 8, 9, 11 years). Control regressors: sex and TIV. B) Subset of neuroimaging cohort 2 matched to cohort 1: regressors of interest: parenting behaviors at according age matched to cohort 1. Control regressors: sex, age, TIV and parental education (SES) for models with significant associations. TIV = total intracranial volume; SES = Parental education used as a proxy for socioeconomic status.

**References**

1. Ribeaud D, Murray A, Shanahan L, Shanahan MJ, Eisner M. Cohort Profile: The Zurich Project on the Social Development from Childhood to Adulthood (z-proso). *J Dev Life Course Criminology*. 2022;8(1):151-171. doi:10.1007/s40865-022-00195-x

2. Frick PJ. *The Alabama Parenting Questionnaire.* 1991.

3. Essau CA, Sasagawa S, Frick P. Psychometric Properties of the Alabama Parenting Questionnaire. *Journal of Child and Family Studies*. 2006;15:595-614. doi:10.1007/s10826-006-9036-y

4. Shelton KK, Frick PJ, Wootton J. Assessment of parenting practices in families of elementary school-age children. *Journal of Clinical Child Psychology*. 1996;25:317-329. doi:10.1207/s15374424jccp2503_8

5. Dale AM, Fischl B, Sereno MI. Cortical surface-based analysis. I. Segmentation and surface reconstruction. *Neuroimage*. 1999;9(2):179-194. doi:10.1006/nimg.1998.0395

6. Fischl B, Sereno MI, Dale AM. Cortical surface-based analysis. II: Inflation, flattening, and a surface-based coordinate system. *Neuroimage*. 1999;9(2):195-207. doi:10.1006/nimg.1998.0396

7. Desikan RS, Ségonne F, Fischl B, et al. An automated labeling system for subdividing the human cerebral cortex on MRI scans into gyral based regions of interest. *Neuroimage*. 2006;31(3):968-980. doi:10.1016/j.neuroimage.2006.01.021

8. Kok R, Thijssen S, Bakermans-Kranenburg MJ, et al. Normal Variation in Early Parental Sensitivity Predicts Child Structural Brain Development. *Journal of the American Academy of Child & Adolescent Psychiatry*. 2015;54(10):824-831.e1. doi:10.1016/j.jaac.2015.07.009

9. Narita K, Takei Y, Suda M, et al. Relationship of parental bonding styles with gray matter volume of dorsolateral prefrontal cortex in young adults. *Progress in Neuro-Psychopharmacology and Biological Psychiatry*. 2010;34(4):624-631. doi:10.1016/j.pnpbp.2010.02.025

10. Yang J, Wei D, Wang K, Yi Z, Qiu J. Regional gray matter volume mediates the relationship between maternal emotional warmth and gratitude. *Neuropsychologia*. 2018;109:165-172. doi:10.1016/j.neuropsychologia.2017.12.017

11. Dimanova P, Borbás R, Raschle N. From Mother to Child: How Intergenerational Transfer Is Reflected in Similarity of Corticolimbic Brain Structure and Mental Health. *PsyArXiv*. Preprint posted online May 22, 2023. doi:10.31234/osf.io/fdphk

12. Fehlbaum LV, Peters L, Dimanova P, et al. Mother-child similarity in brain morphology: A comparison of structural characteristics of the brain’s reading network. *Developmental Cognitive Neuroscience*. 2022;53:101058. doi:10.1016/j.dcn.2022.101058

13. R Core Team. R: A Language and Environment for Statistical Computing. Published online 2022. https://www.R-project.org/

14. RStudio Team. RStudio: Integrated Development Environment for R. Published online 2022. http://www.rstudio.com/

15. van Buuren S, Groothuis-Oudshoorn K. mice: Multivariate Imputation by Chained Equations in R. *J Stat Soft*. 2011;45(3):1-67. doi:10.18637/jss.v045.i03

16. Harrell Jr. FE, Dupont C. Hmisc: Harrell Miscellaneous. R package. Published online 2022. https://CRAN.R-project.org/package=Hmisc

17. Derogatis LR. *Brief Symptom Inventory: Administration, Scoring, and Procedures Manual*. National Computer Systems; 1993.

18. Cohen S, Kamarck T, Mermelstein R. A global measure of perceived stress. *Journal of Health and Social Behavior*. 1983;24(4):385-396. doi:10.2307/2136404

19. Gross JJ, John OP. Individual Differences in Two Emotion Regulation Processes: Implications for Affect, Relationships, and Well-Being. *Journal of Personality and Social Psychology*. 2003;85(2):348-362. doi:10.1037/0022-3514.85.2.348

20. Groeben N, Perren S, Stadelmann S, von Klitzing K. *Inventar Zur Diagnostik Sozialer Kompetenzen (IDS)*. Hogrefe; 2011.

21. Goodman R. The Strengths and Difficulties Questionnaire: A Research Note. *Journal of Child Psychology and Psychiatry*. 1997;38(5):581-586. doi:10.1111/j.1469-7610.1997.tb01545.x

22. Achenbach TM, Rescorla LA. Achenbach, T.M., & Rescorla, L.A. (2001). Manual for the ASEBA School-Age Forms & Profiles. Burlington, VT: University of Vermont, Research Center for Children, Youth, and Families. *Pediatrics In Review*. 2001;21(8). doi:10.1542/pir.21-8-265

23. z-proso Project Team. *Z-Proso Handbook: Instruments and Procedures in the Parent Surveys (Waves P1–P4; 2004–2008)*. Jacobs Center for Productive Youth Development, University of Zurich; 2025.

24. z-proso Project Team. z-proso Handbook: Instruments and Procedures in the Adolescent and Young Adult Surveys (Age 11 to 24; Waves K4-K9). Published online 2024. doi:10.5167/UZH-253680

25. Goldberg DP. Manual of the General Health. In: NELSON Publishers; 1978:8-12.

26. Grasmick HG, Tittle CR, Bursik RJ, Arneklev BJ. Testing the Core Empirical Implications of Gottfredson and Hirschi’s General Theory of Crime. *Journal of Research in Crime and Delinquency*. 1993;30(1):5-29. doi:10.1177/0022427893030001002

27. Tremblay RE, Loeber R, Gagnon C, Charlebois P, Larivée S, LeBlanc M. Disruptive boys with stable and unstable high fighting behavior patterns during junior elementary school. *J Abnorm Child Psychol*. 1991;19(3):285-300. doi:10.1007/BF00911232

28. Dadds MR, Powell MB. The relationship of interparental conflict and global marital adjustment to aggression, anxiety, and immaturity in aggressive and nonclinic children. *J Abnorm Child Psychol*. 1991;19(5):553-567. doi:10.1007/BF00925820

29. Spanier GB. Measuring dyadic adjustment: New scales for assessing the quality of marriage and similar dyads. *Journal of Marriage and the Family*. 1976;38(1):15-28. doi:10.2307/350547

30. Wikström POH, Butterworth D. Adolescent Crime: Individual Differences and Lifestyles. *Adolescent Crime: Individual Differences and Lifestyles*. Published online January 1, 2013:1-286. doi:10.4324/9781843925712

31. Ribeaud D, Eisner M. Risk factors for aggression in pre-adolescence: Risk domains, cumulative risk and gender differences - Results from a prospective longitudinal study in a multi-ethnic urban sample. *European Journal of Criminology*. 2010;7(6):460-498. doi:10.1177/1477370810378116

32. Statistics UIf. International standard classification of education: ISCED 2011. *Comparative Social Research*. Published online 2012.
